# Transcriptional Control of Drug Resistance, Virulence and Immune System Evasion in Pathogenic Fungi: A Cross-Species Comparison

**DOI:** 10.3389/fcimb.2016.00131

**Published:** 2016-10-20

**Authors:** Pedro Pais, Catarina Costa, Mafalda Cavalheiro, Daniela Romão, Miguel C. Teixeira

**Affiliations:** ^1^Biological Sciences Research Group, Department of Bioengineering, Instituto Superior Técnico, Universidade de LisboaLisbon, Portugal; ^2^Biological Sciences Research Group, Institute for Bioengineering and Biosciences, Instituto Superior TécnicoLisboa, Portugal

**Keywords:** transcription factor, antifungal drug resistance, pathogenesis, immune system evasion, *Candida* species, *Cryptococcus neoformans*, *Aspergillus fumigatus*

## Abstract

Transcription factors are key players in the control of the activation or repression of gene expression programs in response to environmental stimuli. The study of regulatory networks taking place in fungal pathogens is a promising research topic that can help in the fight against these pathogens by targeting specific fungal pathways as a whole, instead of targeting more specific effectors of virulence or drug resistance. This review is focused on the analysis of regulatory networks playing a central role in the referred mechanisms in the human fungal pathogens *Aspergillus fumigatus, Cryptococcus neoformans, Candida albicans, Candida glabrata, Candida parapsilosis*, and *Candida tropicalis*. Current knowledge on the activity of the transcription factors characterized in each of these pathogenic fungal species will be addressed. Particular focus is given to their mechanisms of activation, regulatory targets and phenotypic outcome. The review further provides an evaluation on the conservation of transcriptional circuits among different fungal pathogens, highlighting the pathways that translate common or divergent traits among these species in what concerns their drug resistance, virulence and host immune evasion features. It becomes evident that the regulation of transcriptional networks is complex and presents significant variations among different fungal pathogens. Only the oxidative stress regulators Yap1 and Skn7 are conserved among all studied species; while some transcription factors, involved in nutrient homeostasis, pH adaptation, drug resistance and morphological switching are present in several, though not all species. Interestingly, in some cases not very homologous transcription factors display orthologous functions, whereas some homologous proteins have diverged in terms of their function in different species. A few cases of species specific transcription factors are also observed.

## Introduction

Infections caused by fungal pathogens have become a relevant threat to human health as their prevalence has continuously increased over the past decades (Perlroth et al., 2007; Miceli et al., 2011; Miceli and Lee, 2011). Three genera are particularly significant as human pathogens: fungi belonging to the *Aspergillus* spp. and yeasts from *Cryptococcus* spp. and *Candida* spp. Infections caused by these pathogens are especially severe in immunocompromised patients, particularly HIV-infected, cancer and transplant patients (Sims et al., 2005; Chauhan et al., 2006; Pongpom et al., 2015; Schmalzle et al., 2016).

Infection niches and mechanisms diverge according to specific traits of each organism. Infections by *Aspergillus fumigatus* start in the pulmonary epithelia and evolve into allergic bronchopulmonary aspergillosis, aspergilloma, invasive pulmonary aspergillosis and hematogenously disseminated aspergillosis (Brown and Goldman, 2016). On the other hand, infections by the pathogenic yeast *Cryptococcus neoformans* are primarily pulmonary, persisting for long periods of time, and then spreading to the central nervous system (CNS) (Hole and Wormley, 2016). On the contrary, infections by *Candida* spp. are established in mucosal and cutaneous surfaces, translating into systemic infections with high tissue burden if able to invade and reach the bloodstream (Pfaller and Diekema, 2007; Azie et al., 2012; Montagna et al., 2013; Papon et al., 2013).

The severity of infections caused by fungal pathogens is associated with a concerted interplay between antifungal drug resistance, virulence and immune system evasion features. Therefore, it is pertinent not only to study the referred mechanisms, but also the transcriptional networks controlling such traits. This knowledge is crucial to identify new therapeutic targets, while ultimately helping to overcome fungal infections.

This review will focus on the transcriptional regulation of antifungal drug resistance, virulence and immune system evasion mechanisms employed by *Aspergillus fumigatus, Cryptococcus neoformans* and the four most prevalent *Candida* species: *Candida albicans, Candida glabrata, Candida parapsilosis*, and *Candida tropicalis*. This comprehensive analysis aims to identify and compare conserved transcriptional regulators among the indicated organisms, while also contributing to find additional uncharacterized homologs, whose functional analysis may bring further light to these multifactorial processes. This inter-species comparison will provide a better understanding of the regulatory networks applied by fungal pathogens to regulate crucial features for their pathogenic nature and of their variability and evolution among the considered species.

Transcription factors described as relevant regulators of drug resistance, virulence traits and host immune evasion among *A. fumigatus, C. neoformans*, and *Candida* spp. were selected and used to study the variability of regulatory networks governing these processes. Resorting to the Phylome Database (http://phylomedb.org/), the phylomes of each transcription factor were then used to search for protein phylogenies with the objective of identifying predicted homologs in the remaining species. BLASTp analysis was used to complement the Phylome DB data by searching for homologous sequences in the studied species (considering as threshold *E* < 10^−50^). The amino acid sequences of the studied transcriptional regulators in *A. fumigatus, C. neoformans* and *Candida* spp. were retrieved from the *Aspergillus* Genome Database (http://www.aspergillusgenome.org/), *Cryptococcus neoformans* TF Phenome Database (http://tf.cryptococcus.org/), *Candida* Genome Database (http://www.candidagenome.org/), and EnsemblFungi (http://fungi.ensembl.org/) (for *C. tropicalis*), respectively. For tree representation, the MEGA 7 software (http://www.megasoftware.net/) was used to perform multiple sequence alignments and tree visualization. Combining this bioinformatics approach with already described information for characterized transcription factors and their regulatory targets, an inter-species comparison of the transcriptional networks governing important traits such as drug resistance, virulence and immune evasion in yeast and fungal pathogens is presented in this review.

## Drug resistance transcription regulators

In order to overcome drug resistance it is essential to understand the structure of the transcription networks regulating this phenomenon, as it implies a complex regulatory circuit in order to activate the most appropriate response according to distinct stimuli.

One of the major regulators of drug resistance in *C. glabrata* is the transcription factor Pdr1. *C. glabrata* Pdr1 is a Zn(2)-Cys(6) DNA binding protein responsible for the activation of drug resistance genes, such as the multidrug resistance transporters Cdr1 and Pdh1 (also known as Cdr2), via pleiotropic drug response elements (PDRE) (Vermitsky et al., 2006; Caudle et al., 2011; Paul et al., 2011). Gain-of-Function (GOF) mutations in the CgPdr1 transcription factor have been found in clinical isolates to be responsible for increased CgPdr1 activity and consequent constitutive high expression of the ABC drug efflux pumps, as well as its positive autoregulation (Tsai et al., 2006; Ferrari et al., 2009; Paul et al., 2011). Although typical regulatory targets of Pdr1 include the ATP-Binding Cassette (ATP) efflux pumps Cdr1 and Pdh1, it was also found to activate the expression of efflux pumps from the Major Facilitator Superfamily (MFS), including Qdr2 and Tpo3 (Costa et al., 2013, 2014), thus reaffirming its role as a major regulator of drug resistance in *C. glabrata*. No proteins displaying significant homology in the remaining studied species were identified by phylome search.

Drug resistance regulation is a complex process that must be controlled according to the specific stress exerted over fungal pathogens, activating or repressing key pathways to better express the desired response. As such, a negative Zn(2)-Cys(6) regulator of azole resistance, the transcription factor Stb5, was also addressed as a relevant regulator of drug resistance in *C. glabrata*. As a result of its negative regulation, Stb5 overexpression leads to a higher susceptibility toward azole drugs, while its deletion causes a small increase in azole resistance (Noble et al., 2013). Also, expression analysis showed that Stb5 shares many transcriptional targets with Pdr1, such as Cdr1, Pdh1, and Yor1, but working as a pleitropic drug resistance repressor (Noble et al., 2013). Homologous proteins were identified in *C. parapsilosis* (uncharacterized, encoded by *ORF* CPAR2_109760) and in *C. albicans* (Stb5) in the phylome analysis. Additionally, the *C. tropicalis* protein encoded by *ORF CTRG_04421* was identified as a *C. glabrata* Stb5 homolog by BLASTp search. *C. albicans* Stb5 shares the Zn(2)-Cys(6) DNA binding domain found in *C. glabrata* Stb5, and despite the fact that its role and regulation mechanisms are still poorly characterized, it has been shown to be repressed by Hap43 (Singh et al., 2011).

**Figure 1:**
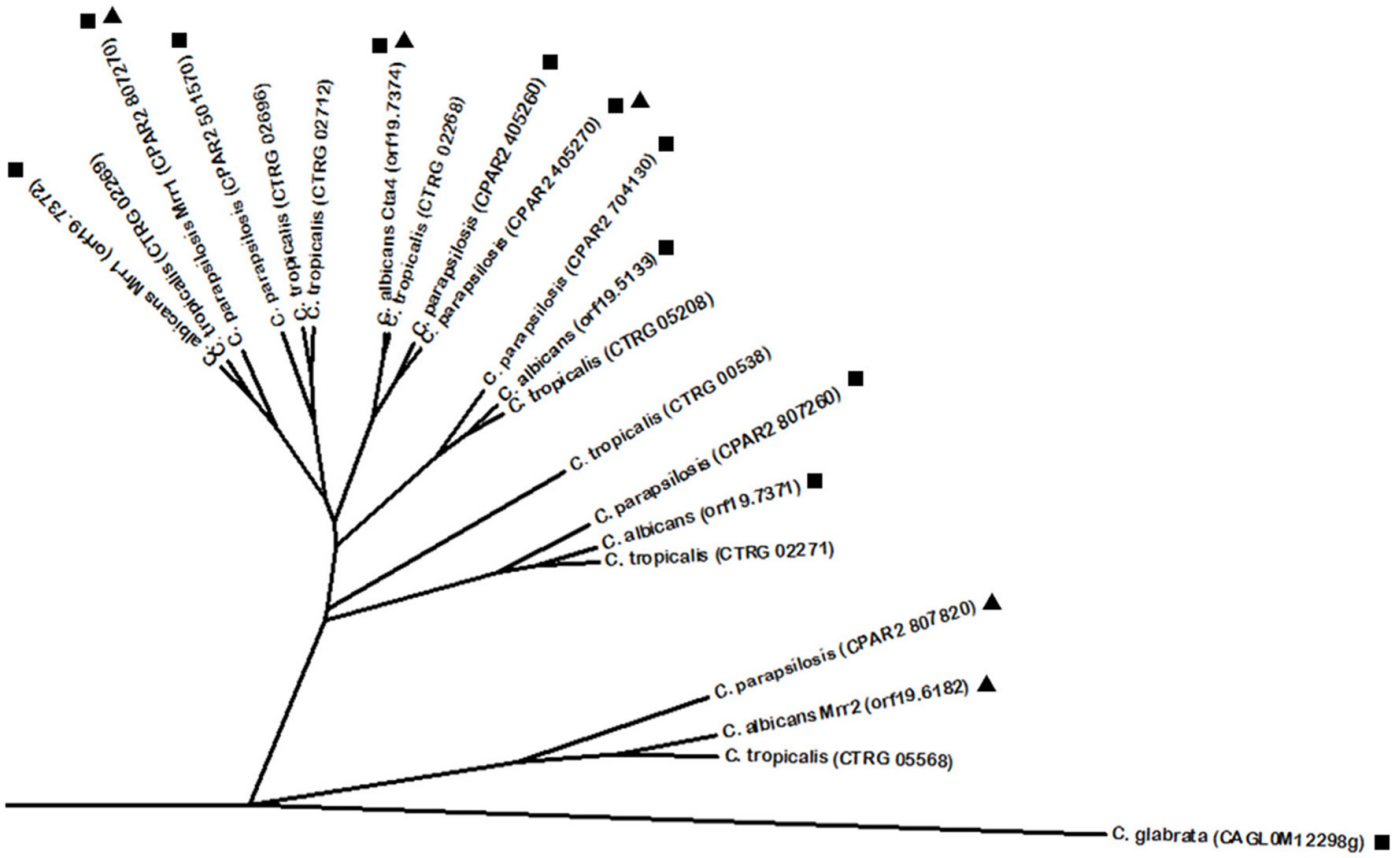
**Phylogenetic analysis of the ***C. albicans*** Mrr1 and Mrr2 homologs**. Phylome predicted homologs of Mrr1 are marked with (■). Phylome predicted homologs of Mrr2 are marked with (▲). Unmarked branches represent additional proteins showing some degree of similarity identified by BLASTp (*E* < 10^−50^). The tree was constructed using the Molecular Evolutionary Genetics Analysis (MEGA 7) software (Kumar et al., 2016). Multiple alignments of the amino acid sequences were calculated by ClustalW algorithm (Sneath and Sokal, 1973). The tree is drawn to scale, with branch lengths in the same units as those of the evolutionary distances used to infer the phylogenetic tree. The evolutionary distances were computed using the JTT matrix-based method (Jones et al., 1992) and are in the units of the number of amino acid substitutions per site. The rate variation among sites was modeled with a gamma distribution (shape parameter = 1).

*C. albicans* has its own master regulator of drug resistance Tac1, a Zn(2)-Cys(6) DNA binding activator of drug-responsive genes such as the ABC multidrug resistance transporters Cdr1 and Cdr2 (Coste et al., 2004) by binding the drug response element (DRE) (Coste et al., 2009). Onward with the transcriptional control of Tac1 over Cdr1 and Cdr2 expression, changes in this transcription factor gene were described to modulate its function and consequently add an extra layer of regulation in its network. Several substitutions and small deletions were found to increase Tac1 function (Coste et al., 2007), while chromosomal rearrangements in chromosome 5 lead to loss of heterozigosity resulting in Tac1 dosage adjustments by overexpression of its encoding gene (Coste et al., 2007; Selmecki et al., 2008). There is evidence supporting positive autoregulation of Tac1 (Liu et al., 2007; Znaidi et al., 2007). Altogether, evidence shows that *TAC1* controls its target genes by increasing its own expression or by GOF mutations (Coste et al., 2006, 2007, 2009). Despite having similar functions and regulating similar gene targets, Tac1 was not found to share sequence homology with *C. glabrata* Pdr1, according to the Phylome database. Instead, Tac1 presents high similarity with several uncharacterized proteins encoded by other CTG clade *Candida* spp. These include *C. parapsilosis* proteins encoded by *ORFs* CPAR2_303510, CPAR2_303520, and CPAR2_303500 and *C. tropicalis* proteins encoded by *ORFs CTRG_05307, CTRG_05306*, and *CTRG_05308*. Interestingly, phylome analysis highlights two other *C. albicans* regulators close to Tac1: the Zn(2)-Cys(6) transcription factors Znc1 and Hal9. These findings are intriguing, given that Znc1 is required for yeast cell adherence to silicone substrate (Finkel et al., 2012) and Hal9 is induced by Mnl1 under weak acid stress (Ramsdale et al., 2008), and therefore do not display a conserved function with Tac1, despite their predicted homology. *C. albicans* carries yet another major regulator of multidrug resistance transporters in the transcription factor Mrr1, an activator of the MFS multidrug transporter Mdr1, leading to acquisition of multidrug resistance in azole resistant clinical isolates (Morschhäuser et al., 2007). As observed for *C. glabrata* Pdr1 and *C. albicans* Tac1 transcriptional regulators, Mrr1 is a Zn(2)-Cys(6) transcription factor and its gene sequence is subjected to GOF mutations responsible for increased protein activity (Dunkel et al., 2008). As described above for Tac1, Mrr1 also appears to be auto-regulated (Schubert et al., 2011). Additionally, it was found to be induced by Hap43 (Singh et al., 2011). Showing some level of functional conservation with the previous regulators is also the transcription factor Mrr2, seen to control the expression of Cdr1 in *C. albicans* (Schillig and Morschhäuser, 2013). Mrr1 and Mrr2 do not present significant homology to each other, but interestingly, in the search for Mrr1 and Mrr2 homologs using PhylomeDB, several common hits were identified in *Candida* spp. (Figure 1). Among them is a closely related *C. parapsilosis* Zn(2)-Cys(6) homolog (named Mrr1) described to be upregulated in azole resistant strains, probably leading to the upregulation of *C. parapsilosis* Mdr1 (Silva et al., 2011). Similarly to what is described in *C. albicans*, the upregulation of *C. parapsilosis* Mrr1 and Mdr1 is correlated with point mutations in the *MRR1* gene (Silva et al., 2011). However, beyond these homologs, BLASTp analysis revealed an array of additional proteins that show some similarity with Mrr1 and Mrr2. Interestingly, *C. parapsilosis* Mrr1 was also found to share sequence similarity with *C. albicans* Mrr2 (Figure 1). Additionally, other regulators not primarily related to drug resistance display sequence similarity with Mrr1 and Mrr2, namely *C. albicans* Cta4 (Coste et al., 2008), a transcription factor involved in nitrosative stress resistance (Chiranand et al., 2008). Nevertheless, it is relevant to point out that Cta4 expression in *S. cerevisiae* was seen to contribute for azole drug resistance (Coste et al., 2008). It is interesting to see that Mrr1 and Mrr2 display some level of similarity not only with other regulators (e.g., Cta4), but also with several uncharacterized proteins, both in *C. albicans* and other *Candida* spp.

**Figure 2:**
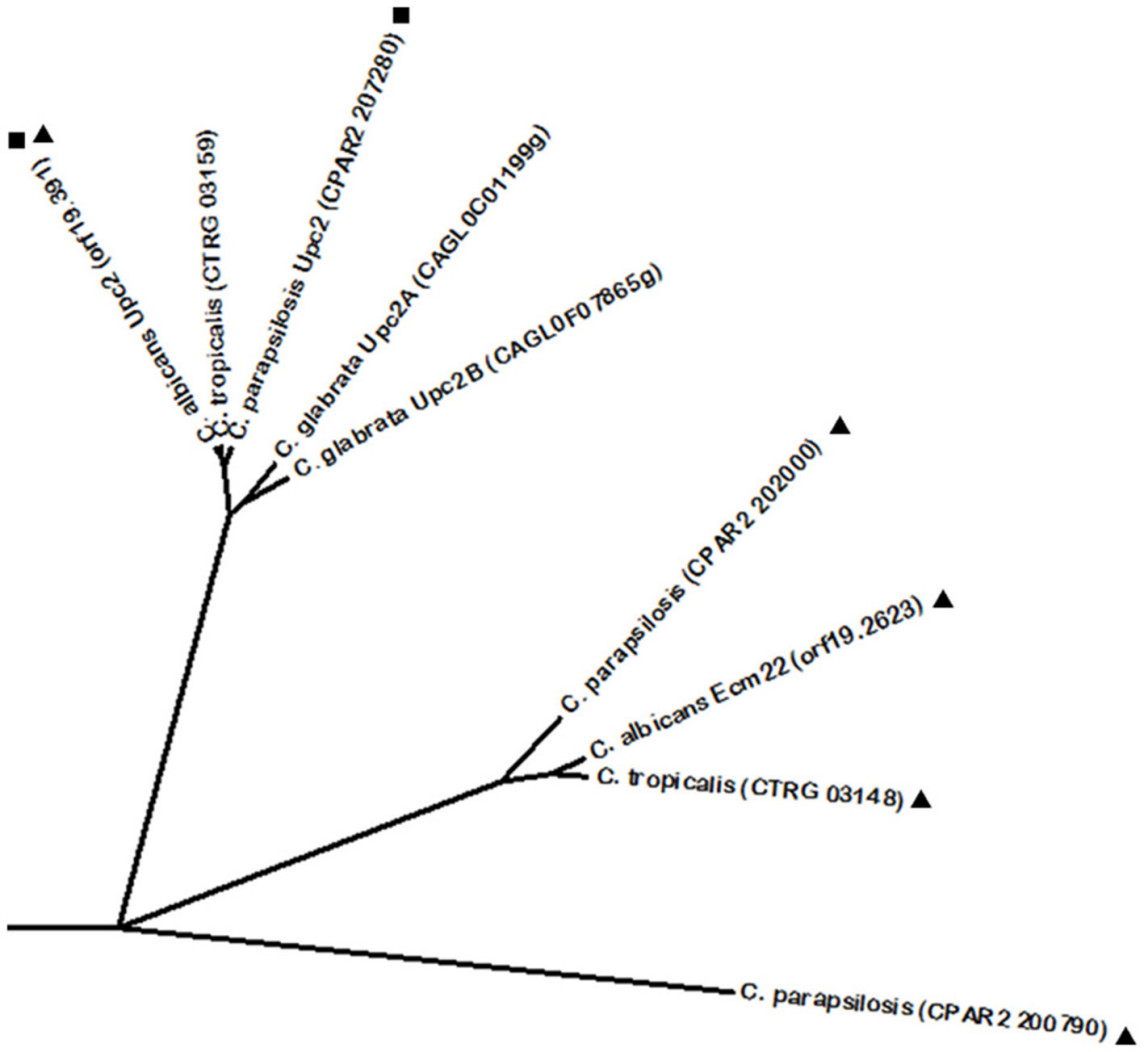
**Phylogenetic analysis of the ***C. albicans*** Upc2 and Ecm22 homologs**. Phylome predicted homologs of Upc2 are marked with (■). Phylome predicted homologs of Ecm22 are marked with (▲). Unmarked branches represent additional proteins showing some degree of similarity identified by BLASTp (*E* < 10^−50^). The tree was constructed using the Molecular Evolutionary Genetics Analysis (MEGA 7) software (Kumar et al., 2016). Multiple alignments of the amino acid sequences were calculated by ClustalW algorithm (Sneath and Sokal, 1973). The tree is drawn to scale, with branch lengths in the same units as those of the evolutionary distances used to infer the phylogenetic tree. The evolutionary distances were computed using the JTT matrix-based method (Jones et al., 1992) and are in the units of the number of amino acid substitutions per site. The rate variation among sites was modeled with a gamma distribution (shape parameter = 1).

Resistance to azole drugs has often been attributed to the upregulation of ergosterol biosynthetic genes, given that these drugs act by inhibiting the activity of Erg11, thus leading to ergosterol depletion in the fungal plasma membrane (Kelly et al., 1995; Ghannoum and Rice, 1999; Kanafani and Perfect, 2008). In this context, the transcription factor Upc2 is an important player in azole drug resistance, being a transcriptional activator of ergosterol biosynthetic genes in *C. albicans*, but also of the MFS multidrug transporter encoding gene *MDR1* (Silver et al., 2004; MacPherson et al., 2005; Dunkel et al., 2008; Heilmann et al., 2010; Synnott et al., 2010). *C. albicans* Upc2 phylome analysis revealed a *C. parapsilosis* Upc2 homolog. In fact, *C. parapsilosis* Upc2 displays a conserved function, as it was described to confer resistance against azole drugs and to regulate the ergosterol pathway in hypoxia (Guida et al., 2011). Despite no other homologs were identified at the Phylome DB, *C. glabrata* harbors two Upc2 regulators known to participate in the same process. *C. glabrata* Upc2A, but not Upc2B, displays the prominent role in the resistance against azoles and sterol biosynthesis inhibitors (Nagi et al., 2011). However, Upc2B was shown to regulate the expression of Erg2 and Erg3 from the ergosterol biosynthetic pathway, whereas both Upc2A and Upc2B are required for expression of the sterol transporter Aus1 (Nagi et al., 2011). Given the conserved role of these proteins with the Upc2 proteins from *C. albicans* and *C. parapsilosis*, their phylogenetic proximity was evaluated using BLASTp analysis, through which a predicted *C. tropicalis* Upc2 was also found to share significant sequence similarity with the rest of the Upc2 proteins (Figure 2). Interestingly, Upc2 phylome analysis did not reveal Ecm22 as a possible homolog; however, reciprocal phylome analysis unveiled Upc2 as an Ecm22-related protein. Several uncharacterized proteins in other CTG clade species were further predicted to share homology with *C. albicans* Ecm22 (Figure 2). Additionally, there is also an identified Ecm22 protein in *C. neoformans*, however, it does not share significant homology with *C. albicans* Ecm22. In most fungi, regulation of sterol biosynthesis is based on well conserved Sterol Regulatory-Element Binding Proteins (SREBP), usually harboring a basic helix-loop-helix (bHLH) leucine zipper domain. However, it is interesting to note that this system has been disrupted in yeasts such as *S. cerevisiae* and *Candida* species (Maguire et al., 2014). In these species, the role of SREBPs in sterol biosynthesis has been replaced by the Zn(2)-Cys(6) Upc2 proteins, which are structurally unrelated to SREBPs. Maguire and colleagues proposed that Upc2 arose in the common ancestor of the Saccharomycotina and was created by duplication of another zinc finger protein gene, although it diverged too much from its orthologs in other species, such as *A. fumigatus* (Maguire et al., 2014).

Upregulation of ergosterol synthesis upon azole drug exposure is controlled in *A. fumigatus* by the transcription factor SrbA, encoding a bHLH protein belonging to the SREBP family. As stated previously, this family comprises the main regulators of sterol biosynthesis in yeasts outside of the Saccharomycotina lineage. SrbA is responsible for mediating azole drug resistance by activating the expression of *cyp51*, the *ERG11* ortholog in this pathogen (Mellado et al., 2005; Willger et al., 2008; Moye-Rowley, 2015). Additionally, it has a secondary role in the maintenance of cell polarity, therefore directing hyphal growth (Willger et al., 2008). SrbA controls the expression of a target gene with which it shares sequence similarity: *srbB*. SrbB is another transcriptional regulator that together with SrbA co-regulates heme biosynthesis and sterol demethylation genes. However, it acquired new functions as it also regulates hypoxia response and virulence genes (Chung et al., 2014). Phylome analysis did not provide any possible SrbA or SrbB homologous proteins in any of the remaining species addressed in this work. Nonetheless, *C. neoformans* harbors the transcription factor Sre1, another bHLH protein that despite not being found to share a phylogenetic relationship with SrbA or SrbB was found to be required for azole drug resistance (Chun et al., 2007; Bien et al., 2009; Jung et al., 2015). Like *A. fumigatus* SrbA, this is due to Sre1 involvement in the expression of genes required for ergosterol biosynthesis (Chang et al., 2007; Willger et al., 2008). Additionally, Sre1 is also involved in virulence, as it was found to be important for the establishment and growth of yeast cells in the brain; as well as being involved in the regulation of genes involved in iron uptake (Chang et al., 2007).

## Virulence transcriptional regulators

### Biofilm formation and tissue invasion regulators

The ability of fungal pathogens to cause disease relies upon an array of strategies to colonize surfaces and invade host tissues. The establishment of biofilms is one of the main virulence traits displayed by human pathogens. The development of biofilms in medical devices represents a relevant risk factor for patients, as such devices serve as reservoirs and entry points for potential infections (Douglas, 2003; Kojic and Darouiche, 2004; Martinez and Casadevall, 2015). Once inside the host, the development of biofilms allows pathogens to overcome environmental stresses, such as drug exposure and immune system attack, while also resulting in the establishment of persistent infections (Jabra-Rizk et al., 2004; Fanning and Mitchell, 2012).

**Figure 3:**
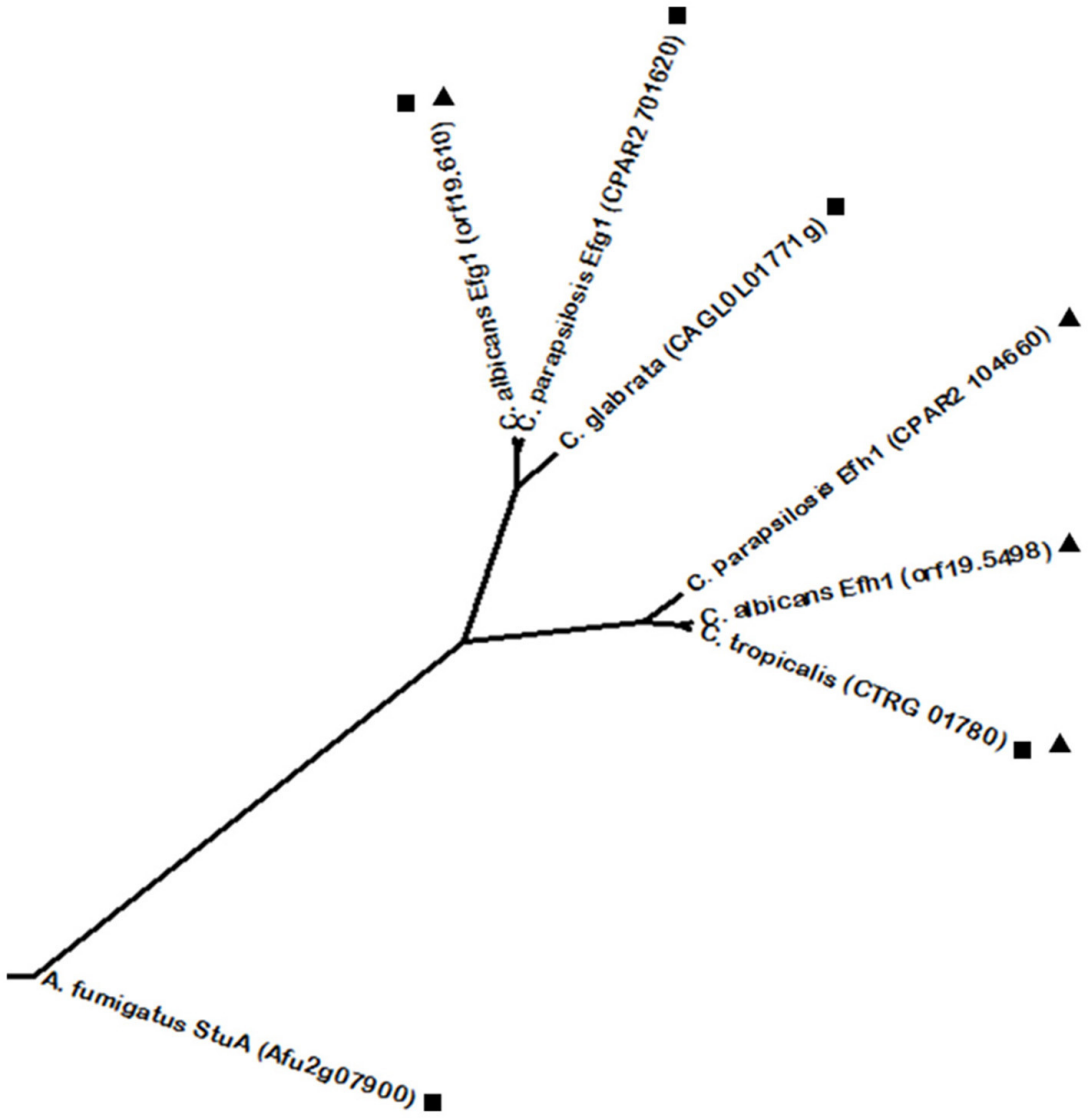
**Phylogenetic analysis of the ***C. albicans*** Efg1 and Efh1 homologs**. Phylome predicted homologs of Efg1 are marked with (■). Phylome predicted homologs of Efh1 are marked with (▲). The tree was constructed using the Molecular Evolutionary Genetics Analysis (MEGA 7) software (Kumar et al., 2016). Multiple alignments of the amino acid sequences were calculated by ClustalW algorithm (Sneath and Sokal, 1973). The tree is drawn to scale, with branch lengths in the same units as those of the evolutionary distances used to infer the phylogenetic tree. The evolutionary distances were computed using the JTT matrix-based method (Jones et al., 1992) and are in the units of the number of amino acid substitutions per site. The rate variation among sites was modeled with a gamma distribution (shape parameter = 1).

Generally, biofilm formation is among the most studied subjects in virulence and is based on adherence and morphogenic phenomena, including hyphae formation. Besides biofilm formation, another key virulence factor is invasion of non-phagocytic host cells, as it represents the ability of the pathogen to disseminate and infect the host. Epithelial cell invasion by *Candida* spp. has been correlated with the production of lytic enzymes, such as secreted aspartyl proteinases (SAPs) that digest the surface of epithelial tissue thus enabling tissue invasion (Schaller et al., 1999, 2003). In the case of *C. albicans*, hyphae development is also associated with non-phagocytic host cell invasion, as Sap4-6 enzymes are regarded as hyphal-associated proteins (Schweizer et al., 2000; Korting et al., 2003) and hyphae are found within epithelial cells, whereas yeast forms are found on their surface or between them (Scherwitz, 1982; Ray and Payne, 1988). For this reason, hyphal form is considered to be the invasive form of *C. albicans*. Additionally, hyphae were also associated with a more efficient induction of epithelial cell endocytosis, a process in which epithelial cells are stimulated to produce pseudopods that internalize the pathogen (Park et al., 2005). Interestingly, the role of hyphae in the invasion of endothelial tissue appears to be more complex, as distinct tissues endocytose preferentially hyphae, while other endothelial cell linings are as easily crossed by yeast cells (Klotz et al., 1983; Jong et al., 2001; Lossinsky et al., 2006). Yeast-to-hyphae transition is a well-studied feature in *C. albicans*. It can be activated by a variety of conditions, mostly by stress factors, such as changes in pH and temperature (e.g., 37°C) and nitrogen starvation that promote hyphal growth (Kadosh and Johnson, 2005). One of the most powerful factors to induce hyphae formation is the presence of serum, as its nutrients are usually unavailable to *C. albicans* cells, thus constituting a stress condition and therefore inducing hyphal growth. The same principle was verified when using N-acetylglucosamine (Glc-NAc), a poor source of carbon and nitrogen capable of inducing hyphae formation (Mattia et al., 1982; Kadosh and Johnson, 2005). The *C. albicans* positive regulators Efg1 and Cph1 regulate a defined core set of genes required for hyphal growth, including *ECE1, HYR1, HWP1*, and *ALS3*. These genes encode mainly cell wall-associated proteins, involved in processes including hyphal-cell elongation and adhesion to host tissues (Birse et al., 1993; Bailey et al., 1996; Staab et al., 1996, 1999; Hoyer et al., 1998). It is understandable that the transcriptional regulators controlling hyphae formation are responsible for the expression of cell wall related genes, given that yeast-hyphae transition implicates morphological alterations that require cell wall remodeling (Chaffin et al., 1998; Sohn et al., 2003). Analyzing the phylogenetic relationship between *C. albicans* transcription factor Efg1 and its closest homologs, the *C. parapsilosis* Efg1 and *A. fumigatus* StuA transcription factors are highlighted (Figure 3). All three homologs contain an APSES DNA binding domain, which contributes to their close relationship. In turn, *C. parapsilosis* Efg1 was found to be a morphological switch regulator, similarly to its *C. albicans* ortholog (Connolly et al., 2013). In turn, *A. fumigatus* StuA controls adhesion and virulence by regulation of the *uge3* gene, encoding for uridine diphosphate (UDP)-glucose-epimerase which is essential for adherence through mediating the synthesis of galactosaminogalactan (Lin et al., 2015). Other than regulating conidiophore morphology, whole genome transcriptional analysis identified StuA as regulating secondary metabolite biosynthesis genes, the catalase gene *cat1* and morphogenesis genes (Sheppard et al., 2005). Interestingly, a *C. glabrata* homolog (encoded by *ORF CAGL0L01771g*) was identified, although *C. glabrata* is unable to develop true hyphae. Furthermore, *C. albicans* Efg1 has a paralog, Efh1. Efh1 is also an APSES protein but with a minor role compared to Efg1, which is also the case of the *C. parapsilosis* Efg1 ortholog (Doedt et al., 2004; Connolly et al., 2013). However, Phylome DB shows that Efh1 homologs are restricted to *Candida* spp., with homologous proteins in both *C. tropicalis* and *C. parapsilosis* (Figure 3). As expected given their paralogous status, *C. albicans* Efg1 and Efh1 were found to be phylogenetically related with each other by phylome analysis. Interestingly, only one single *C. tropicalis* protein (encoded by *ORF CTRG_01780*) was found to be homologous to both *C. albicans* Efg1 and Efh1, while the single *C. glabrata* protein was found to share homology only with Efg1 (Figure 3). Relative to *C. albicans* Cph1, phylome analysis found it to be closely related to the *C. parapsilosis* Cph1 protein. *C. tropicalis* also features a Cph1 protein, despite it was not considered to have a homology relationship at the Phylome DB. Nevertheless, BLASTp shows a high degree of sequence homology between the Cph1 proteins from *C. albicans* and *C. tropicalis*. All Cph1 proteins belong to the STE-like transcription factor family. As observed for the yeast model *S. cerevisiae*, in which the Cph1 ortholog is the Ste12 transcription factor, other species also have Ste12 homologs from Cph1. Accordingly, *C. glabrata* Ste12 was described as being required for nitrogen starvation induced filamentation and to have a role in virulence (Calcagno et al., 2003). Despite its functional conservation, *C. glabrata* Ste12 was not predicted to be a Cph1 homolog by phylome analysis, indicating that their sequences have somewhat diverged. However, BLASTp comparison shows a significant degree of sequence homology between the two proteins. According to the Phylome DB, no homologs were predicted for *C. glabrata* Ste12, nonetheless, BLASTp analysis revealed an additional *C. glabrata* protein, encoded by *ORF CAGL0H02145g*, presenting significant homology. Moreover, *A. fumigatus* contains a SteA protein, also belonging to the STE-like family, that showed significant homology to *C. glabrata* Ste12 by BLASTp analysis, but this transcription factor is still uncharacterized. *C. neoformans* also harbors a Ste12 transcription factor, though it was not found to share an evolutionary link to the other STE-like family proteins in the previously considered species. This fact, together with the knowledge that *C. neoformans* Ste12 is associated with this pathogen's particular trait of capsule formation (Chang et al., 2001) and melanin production through the expression of the *LAC1* gene (Jung et al., 2015), seems to indicate that the presence of a STE-like domain can be the only trait shared with the remaining proteins. Capsule and melanin production in *C. neoformans* are regulated by a cyclic AMP-dependent protein kinase A (Pka) signaling pathway. Pka is composed of a catalytic (Pka1) and a regulatory subunit (Pkr1) (D'Souza et al., 2001). Mutant strains lacking Pka1 do not produce a capsule under normal conditions, whereas disruption of Pkr1 results in capsule overproduction and hypervirulence, providing evidence of the importance of this pathway in *C. neoformans* virulence (D'Souza et al., 2001; D'Souza and Heitman, 2001). This may be explained by the regulation by Pka1 of glucan synthesis-related genes, important for the production of the capsule, such as *FKS1, AGS1, AGN1, KRE6, KRE61*, and *SKN1* (O'Meara and Alspaugh, 2012). This pathway is responsible for the activation of the Ste12α transcription factor, involved in mating, since *pka1* mutants restored a mating phenotype by overexpression of this transcription factor (D'Souza et al., 2001). The Ras signal transduction pathway was also previously shown to be involved in *C. neoformans* virulence (Alspaugh et al., 2000). The virulence of a *C. neoformans ras1* mutant was attenuated and the induction of the RAS pathway and capsule formation have been associated with growth in minimal media (Alspaugh et al., 1997, 2000). Ras1 is a major *C. neoformans* Ras protein found to contribute to high-temperature growth and invasive growth, which are essential features for proliferation inside the host (Alspaugh et al., 2000). The Ras1/Cdc24 and Ras1/Cdc42 pathways are required for thermotolerance and actin cytoskeleton regulation, whereas Ras1/cAMP governs mating and invasive growth (Alspaugh et al., 2000; Waugh et al., 2003; Nichols et al., 2007). Initially, Ras1 absence does not result in defects in capsule or melanin production and the lack of virulence is attributed to lack of growth at 37°C (Alspaugh et al., 2000). Nevertheless, Ras1 was later shown to have a role in capsule formation induced by serum, however this was not considered as a major mechanism through which Ras1 signaling affects virulence (Zaragoza et al., 2003; Haynes et al., 2011). In the case of *C. neoformans*, much less is known about its adhesion and invasion strategies. *In vitro*, it was shown to adhere and be internalized by pulmonary epithelial cells, thus resulting in host cell damage (Barbosa et al., 2006). Upon reaching the brain, *C. neoformans* cells were found to cross endothelial cell lining by transcytosis, however, such process appears to cause minimal cell damage (Chrétien et al., 2002; Chen et al., 2003; Chang et al., 2004). Although not strictly required, the presence of a capsule appears to enhance initial adherence to endothelial cells and the rate of transcytosis (Chen et al., 2003; Chang et al., 2006). However, this effect seems to be dependent on the endothelial tissue in question (Ibrahim et al., 1995). Nevertheless, evidence suggests that *C. neoformans* capsule is bound by a receptor mainly present in brain endothelial cells, thus potentiating brain tissue invasion (Filler and Sheppard, 2006). Another well characterized pathway of biofilm formation is based on the *C. albicans* regulators Tec1 and Bcr1 (Schweizer et al., 2000; Nobile and Mitchell, 2005). Tec1 is a positive regulator of morphogenesis belonging to the TEA/ATSS family that is predominantly expressed during hyphal growth and is required for hyphae formation during serum induction, during macrophage evasion after phagocytosis and for expression of the aspartyl proteinase genes *SAP4-6* (Schweizer et al., 2000). Tec1 is in turn regulated by Efg1 (Lane et al., 2001a). Moreover, Tec1 expression is directly regulated by Cph2 (Lane et al., 2001a,b). Bcr1 is a C_2_H_2_ zinc finger transcriptional activator of cell-surface protein and adhesion genes such as the previously referred *ECE1, ALS3, HWP1*, and *HYR1* (Nobile and Mitchell, 2005; Nobile et al., 2006). Bcr1 was found to relay a signal within the hyphal developmental network, being positively regulated by Tec1 (Nobile and Mitchell, 2005; Nobile et al., 2006). Starting with the analysis of Tec1 phylogenetic relationships, one close homolog was identified in *C. parapsilosis* (encoded by *ORF* CPAR2_805930). Despite not showing a homology relationship according to phylome analysis, the *A. fumigatus* AbaA transcription factor shares the TEA/ATTS domain and also presents a related function. AbaA regulates the specific *A. fumigatus* feature of conidiation by activating the expression of the velvet regulators *veA* and *velB* (Park et al., 2012), but similarly to the role of Tec1, AbaA also controls adherence, a trait that correlates with conidiatian in *A. fumigatus* (Lin et al., 2015). Additionally, AbaA activates the expression of *wetA*, a regulator with a predicted role in hyphal growth (Tao and Yu, 2011). Regarding the phylogenetic relationships of Bcr1, close homologs were identified in the CTG clade species, including *C. parapsilosis* Bcr1, also involved in biofilm formation (Ding and Butler, 2007; Ding et al., 2011) and an uncharacterized *C. tropicalis* homolog (*ORF CTRG_00608*).

Also related with biofilm formation regulation is the *C. glabrata* transcription factor Cst6. It is a bZIP transcription factor involved in the negative regulation of Epa6, the major adhesin found in *C. glabrata* biofilms (Riera et al., 2012). Additionally, Cst6 also accumulates other roles as demonstrated by the control exerted over the carbonic anhydrase Nce103 in response to carbon dioxide (Cottier et al., 2013). Although no close homologs were predicted in the phylome analysis, the Rca1/Cst6 *C. albicans* transcription factor plays a role related to that of *C. glabrata* Cst6, as it was characterized as a regulator of hyphal formation through the transcription factor Efg1 and positive control of hyphal genes including *GWP1, ECE1, HGC1*, and *ALS3* (Vandeputte et al., 2012). Additionally, it was also found to control CO_2_ sensing by regulating the expression of the carbonic anhydrase Nce103 (Cottier et al., 2012) and antifungal drug resistance (negative regulation of azole and echinocandin drug response, whereas positive regulation of 5-flucytosine response), associated to the regulatory control of cell wall genes (Vandeputte et al., 2012). Additionally, reciprocal phylome analysis of Rca1 identified one predicted homolog in *C. parapsilosis* (*ORF* CPAR2_109540). Furthermore, BLASTp analysis revealed an additional *C. tropicalis* protein (encoded by *ORF CTRG_04281*) showing high degree of homology with *C. albicans* Rca1.

Similarly to what is observed in *Candida, A. fumigatus* conidia (yeast form cells) and hyphae are known to induce their own endocytosis by alveolar epithelial cells through pseudopod engulfment (DeHart et al., 1997; Paris et al., 1997; Zhang et al., 2005). Hyphae development of *A. fumigatus* in alveolar cells occurring after internalization results in no detectable damage to the host cell (Wasylnka and Moore, 2003). Interestingly, conidia endocytosis was also found to result in pneumocyte apoptosis inhibition, therefore showing the importance of host cell invasion in *A. fumigatus* infections (Berkova et al., 2006). The pulmonary epithelium is also penetrated by hyphae, contributing to the subsequent invasion of endothelial tissue by passing from the abluminal to the luminal surface of endothelial cells or by hyphae fragments that enter the bloodstream and disseminate to other organs by invading endothelial cells (Filler and Sheppard, 2006). More recently, a key regulator of biofilm formation in *A. fumigatus*, SomA, was identified (Lin et al., 2015). SomA controls conidiation primarily by acting in the expression of *flbB*, a bZIP transcription factor which controls the expression of other regulatory genes such as *brlA, medA*, and *stuA*, thus having a central role in the network regulating biofilm formation and adherence in *A. fumigatus* (Lin et al., 2015). SomA also takes part on the regulation of *uge3* expression (previously referred) and the spore hydrophobin RodA, which provides adherence (Thau et al., 1994; Lin et al., 2015). Phylome analysis did not reveal any protein in the remaining species addressed in this study that shares significant homology with *A. fumigatus* SomA. As stated previously, one of the regulatory genes controlled by SomA is the transcription factor *brlA*, encoding a C_2_H_2_ zinc finger protein that represents a central regulator for the asexual development and controls the formation of vesicles required for conidiation processes (Lin et al., 2015). BrlA induces the expression of the previously referred *abaA* and *wetA* regulatory genes, which induce differentiation of spore forming cells and the subsequent maturation of conidia (Yu, 2010). *A. fumigatus* MedA is another transcription factor regulated by SomA that together with it regulates BrlA expression. As a result, MedA is a positive regulator of conidiation (Adams et al., 1988). Interestingly, BLASTp analysis revealed a *C. neoformans* protein, encoded by *ORF CNAG_03859*, with high homology to MedA. Together with StuA (also regulated by SomA), MedA regulates adhesion and virulence in *A. fumigatus* by regulation of the *uge3* gene (Lin et al., 2015).

Involved in the regulation of biofilm formation is also the *C. neoformans* Znf2 transcription factor. This transcription factor contains a C_2_H_2_ zinc finger domain and is responsible for control of filamentation, but also of the expression of an important adhesin in *C. neoformans*, Cfl1 (Wang et al., 2012). This adhesin is involved in cell adhesion and biofilm formation. Searching for possible Znf2 homologs using the Phylome DB, the *A. fumigatus* ZafA transcription factor was identified. Interestingly, ZafA has acquired a distinct function in *A. fumigatus*: it is a zinc-responsive regulator, found to be required for *A. fumigatus* virulence by regulating zinc homeostasis (Moreno et al., 2007). The *C. albicans* transcription factor Csr1 was also found to share similarity with Znf2. It shares the C_2_H_2_ zinc finger domain and is also involved in filamentous growth regulation by regulating the expression of, for instance, *HWP1* (Kim et al., 2008; Nobile et al., 2009; Finkel et al., 2012), therefore showing not only sequence similarity but also functional conservation. In turn, Csr1 phylome analysis unveiled homologous proteins in *C. parapsilosis, C. tropicalis*, and *C. glabrata* encoded by *ORFs* CPAR2_403080, *CTRG_03883*, and *CAGL0J05060g*, respectively.

The transcriptional regulation of biofilm formation is complex, being dependent on a diversity of environmental conditions. As a result, biofilm regulatory networks also feature negative regulators that ensure a tight control of this process. Two of the most well characterized negative regulators of biofilm formation are the *C. albicans* regulators Nrg1 and Rfg1 (Braun et al., 2001; Khalaf and Zitomer, 2001; Murad et al., 2001a). Nrg1 is a C_2_H_2_ zinc finger transcription factor that acts together with the general corepressor Tup1 to suppress hyphal growth and expression of hypha-specific genes, which are derepressed as a result of Nrg1 downregulation in typical filamentation conditions (Braun et al., 2001; Murad et al., 2001a,b; Kadosh and Johnson, 2005). Nrg1 also represses the expression of chlamydospore formation genes, by repressing *CSP1* and *CSP2*, two specific chlamydospore related genes (Palige et al., 2013). As for Rfg1, it is a HMG domain negative regulator of several genes that were previously induced by filamentation inducing conditions, indicating that this transcription factor is required for hyphae derepression even under such stimuli (Kadosh and Johnson, 2005). Both *RGF1* and *NRG1* negatively regulate the expression of the hyphae-specific genes *ALS3, ECE1*, and *HWP1*, however, their regulons do not completely overlap (Kadosh and Johnson, 2001, 2005), indicating a distinct function of each regulator in control of hyphae formation in *C. albicans*. It is noteworthy to point out that Nrg1 negatively regulates another transcription factor, *Ume6*, required for hyphal extension, which is also associated with virulence (Banerjee et al., 2008, 2013). Analyzing the phylogenetic relationships of Nrg1 with its homologous proteins, one identified homolog was *C. tropicalis* Nrg1, sharing the C_2_H_2_ zinc finger domain and with a conserved role in filamentation repression (Zhang et al., 2016). Additional uncharacterized homologs were found in *C. parapsilosis, C. tropicalis* and *C. glabrata*, encoded by *ORFs* CPAR2_300790, *CTRG_00608*, and *CAGL0G08107g*, respectively. *C. neoformans* also harbors a Nrg1 protein, conserving the C_2_H_2_ domain but with a more specialized role, as Nrg1 was found to be an activator of capsule formation in *C. neoformans* (O'Meara and Alspaugh, 2012). This specialized function can be the result of a divergent phylogenetic relationship, which could justify why it was not identified as a homolog of *C. albicans* Nrg1 in phylome analysis. *C. neoformans* Nrg1 was found to be responsible for capsule formation since mutants in its encoding gene showed a defect in capsule induction. This transcription factor is activated downstream of the cAMP-PKA cascade (O'Meara and Alspaugh, 2012). For the case of Rfg1, phylome analysis only revealed one homolog, an uncharacterized *C. parapsilosis* protein, encoded by *ORF* CPAR2_801100.

The *C. glabrata* transcription factor Ace2 was found to be a negative regulator of virulence in this pathogenic yeast since its inactivation leads to an increase in the ability of *C. glabrata* to cause disease by almost 200-fold (Kamran et al., 2004), thus being regarded as a major virulence regulator in this yeast. Ace2 was also found to regulate the expression of *CTS1, EGT2, TAL1*, and *TDH3* genes, involved in cell separation and biofilm formation processes, which may be related with the hypervirulence phenotype (Stead et al., 2010). Searching for possible homologs, an additional *C. glabrata* protein, Swi5, was identified by phylome analysis. Swi5 is mostly uncharacterized, but it appears to have a conserved function, given that Swi5 mutants display increased fungal burdens in mouse lungs and brain (MacCallum et al., 2006). Despite not being identified by phylome analysis, *C. albicans* also contains an Ace2 transcription factor; however, its sequence appears to have diverged too much for a phylogenetic relationship to be fully established. Nevertheless, it conserves the C_2_H_2_ zinc finger domain as well as a related role in regulation of a wide variety of pathways in *C. albicans*, including regulation of morphogenesis, cell separation, adherence and virulence (Kelly et al., 2004). Furthermore, Ace2 appears to play distinct functions in the regulation of such traits: its absence results in hyperfilamentation and hypervirulence (Kelly et al., 2004; MacCallum et al., 2006), however, it was found to be required for filamentous growth under hypoxic conditions (Mulhern et al., 2006) and to act as positive regulator of biofilm formation during normoxia (Stichternoth and Ernst, 2009). Related with these roles, it was found to be a positive regulator of the cell wall genes *DSE1* and *SCW11* (Kelly et al., 2004). Additionally, it also plays a role as regulator of antifungal drug resistance against antimycin A (Stichternoth and Ernst, 2009). Concordantly, a *C. parapsilosis* Ace2 homolog conserves the C_2_H_2_ domain and was found to be a biofilm regulator (Holland et al., 2014). BLASTp analysis unveiled yet another Ace2 homolog in *C. tropicalis* encoded by *ORF CTRG_03073*. Despite not sharing significant homology, *A. fumigatus* also harbors an Ace2 protein, sharing the C_2_H_2_ zinc finger domain. As the remaining regulators, *A. fumigatus* Ace2 is involved in the regulation of several mechanisms, ranging from conidiophore development, pigment production and virulence (Ejzykowicz et al., 2009). Additionally, the lack of Ace2 results in increased invasion capacity and virulence, translated into increased pulmonary fungal burden. The higher virulence phenotype is related with Ace2 control over *ppoC, ecm33*, and *ags3* expression (Ejzykowicz et al., 2009).

### Host adaptation regulators

Despite the ability to adhere and form biofilms, there is a much larger set of features that determines the degree of damage caused by a pathogen upon infecting the host. Such traits can be conserved among fungal pathogens, or they can be specific according to the specific characteristics of each pathogen. One general virulence factor is the ability to metabolize available sources of nitrogen. Nitrogen source utilization affects morphological transitions and virulence factor production that confer a competitive advantage for survival, proliferation and colonization (Lee et al., 2013; Ene et al., 2014). One conserved family of nitrogen utilization transcriptional regulators is the Gat1 family. The best characterized is Gat1 from *C. albicans*. It is a transcription factor involved in nitrogen catabolite repression and utilization of isoleucine, tyrosine and tryptophan as sole nitrogen sources (Limjindaporn et al., 2003). Accordingly, *C. albicans* Gat1 regulates the expression of nitrogen associated genes, including *GAP1, UGA4, DAL5*, and *MEP2* (Limjindaporn et al., 2003; Dabas and Morschhäuser, 2007). Phylome analysis reveals a Gat1 homolog in *C. parapsilosis*. Interestingly, *A. fumigatus* transcription factor AreA, which does not display sequence homology to *C. albicans* Gat1, is similarly involved in nitrogen catabolite repression and nitrate utilization, also showed to contribute to virulence (Hensel et al., 1998; Lamarre et al., 2008). Curiously, *C. neoformans* also expresses a Gat1 protein, but it was not found to have a role in virulence, according to a phenotypic screening (Jung et al., 2015), and was not found to be phylogenetically related to Gat1. Subsequent BLASTp search showed the existence of a *C. tropicalis* homolog encoded by *ORF CTRG_03831*. Nevertheless, the presence of Gat1 proteins is markedly conserved among fungal pathogens, which is specially reinforced by the conservation of the GATA DNA binding domain present in these proteins.

Another general feature that correlates with several virulence traits is the ability to activate different cellular pathways in response to pH changes. Rim101 from *C. albicans* is known to regulate the induction of alkaline expressed genes and repress acid expressed genes at alkaline pH (Ramon et al., 1999). Additionally, it is part of the regulatory circuit that control hyphae stimulation in response to alkaline pH (Davis et al., 2000). Phylome analysis predicts one closely related homolog in *C. parapsilosis*, encoded by *ORF* CPAR2_700450. Furthermore, BLASTp indicates another homolog in *C. tropicalis*, Rim101. *C. neoformans* also has an identified Rim101 regulator, but interestingly, it was found to regulate capsule maintenance (O'Meara and Alspaugh, 2012), thus showing some level of specialization in this pathogen and reinforcing the hypothesis of a divergent evolution when compared to the remaining Rim101 proteins. Additionally, *C. neoformans* Rim101 is activated after phosphorylation by Pka1 (O'Meara et al., 2010). Low nitrogen and glucose concentration are also inducers of capsule formation by activation of the cAMP pathway. Additionally, the *A. fumigatus* transcription factor PacC displays a similar role in this fungus, once it also regulates alkaline responsive genes, including the dehydrin-like *dprB* (Wong Sak Hoi et al., 2011; Brown and Goldman, 2016). Furthermore, it was found to play a role in host invasion capacity (Bertuzzi et al., 2014) and is also involved in cell wall biogenesis (Brown and Goldman, 2016). Likely, all proteins contain a C_2_H_2_ zinc finger domain that is conserved among all species. Interestingly, the *S. cerevisiae* Rim101 was found to play an additional role in weak acid stress tolerance (Mira et al., 2009).

Following the same principle, adaptation to weak acid stress is another relevant factor in the establishment of infection, especially in niches where such conditions are felt, as in the vaginal tract. One characterized transcriptional regulator of weak acid resistance resistance is *C. albicans* War1, a Zn(2)-Cys(6) transcription factor required for resistance to weak organic acids such as sorbate (Lebel et al., 2006). It acts similarly to the *S. cerevisiae* War1 protein that governs weak acid stress response (Schüller et al., 2004). Looking for War1 homologs in other fungal pathogens, phylome analysis shows homologs in each of the CTG clade *Candida* spp. (encoded by *ORFs* CPAR2_110360 and *CTRG_04350* in *C. parapsilosis* and *C. tropicalis*, respectively), closely related to that of *C. albicans*. Additionally, one War1 homolog was also identified in *C. glabrata*, uncharacterized until now. Additionally, no War1 homologs were found in *C. neoformans*, while two *A. fumigatus* uncharacterized homologs were identified (encoded by *ORFs Afu7g01640* and *Afu8g00950*).

**Figure 4:**
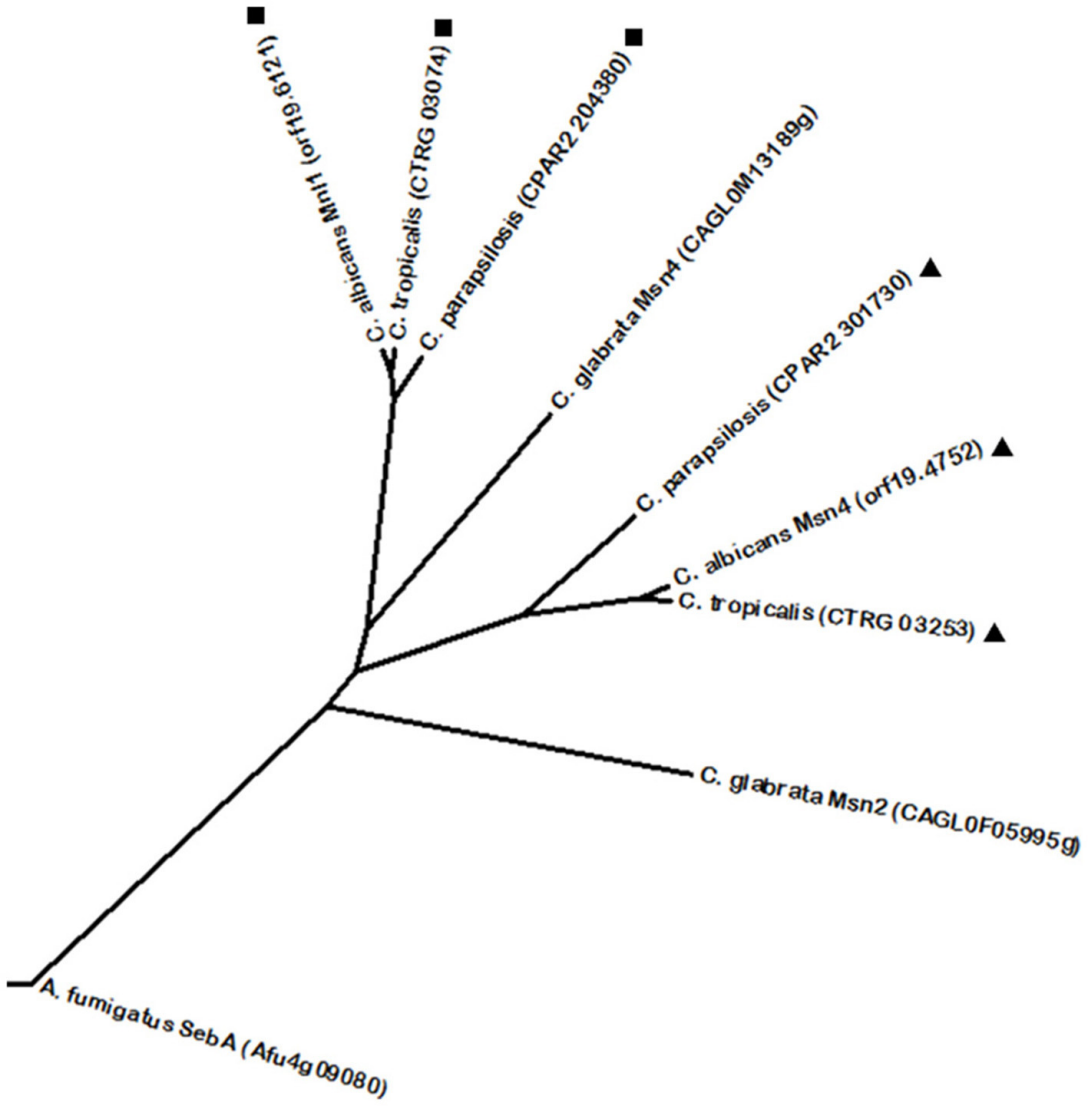
**Phylogenetic analysis of the ***C. albicans*** Mnl1 and Msn4 homologs**. Phylome predicted homologs of Mnl1 are marked with (■). Phylome predicted homologs of Msn4 are marked with (▲). Unmarked branches represent additional proteins showing some degree of similarity identified by BLASTp (*E* < 10^−50^). The tree was constructed using the Molecular Evolutionary Genetics Analysis (MEGA 7) software (Kumar et al., 2016). Multiple alignments of the amino acid sequences were calculated by ClustalW algorithm (Sneath and Sokal, 1973). The tree is drawn to scale, with branch lengths in the same units as those of the evolutionary distances used to infer the phylogenetic tree. The evolutionary distances were computed using the JTT matrix-based method (Jones et al., 1992) and are in the units of the number of amino acid substitutions per site. The rate variation among sites was modeled with a gamma distribution (shape parameter = 1).

One of the most well characterized regulators of weak acid stress response in fungal pathogens is *C. albicans* Mnl1. It is a Zn(2)-Cys(6) transcription factor required for adaptation to weak acid stress, activating a subset of genes that are repressed by the previously mentioned Nrg1, through SLE (STRE-like) elements (Hope et al., 2004; Ramsdale et al., 2008). Mnl1 is considered to be related with the yeast conserved Msn2/4 proteins. In fact, *C. albicans* Mnl1 is also known as the Msn2 correspondent in this yeast. Similar to *S. cerevisiae* Msn2/4 that are involved in the general stress response, *C. albicans* Mnl1 is required for the induction of stress response genes via SLE elements (Martínez-Pastor et al., 1996; Ramsdale et al., 2008). Mnl1 phylome analysis revealed homologous proteins in the other CTG clade species (Figure 4). Furthermore, *C. albicans* also features a Msn4 protein. Similarly to Mnl1, phylome analysis shows Msn4 homologs in the remaining CTG clade species (Figure 4), however, Msn4 does not appear to be a significant stress response regulator, unlike its *S. cerevisiae* homolog (Nicholls et al., 2004), and was found to be induced during biofilm formation (Nobile and Mitchell, 2005). Interestingly, despite the fact that *C. glabrata* harbors Msn2/4 proteins involved in oxidative stress resistance by activating the expression of the catalase gene *CTA1* (Cuéllar-Cruz et al., 2008), these regulators were not found to share a phylogenetic relationship with its *C. albicans* counterparts. Likewise, *A. fumigatus* SebA is a transcription factor described to be involved in response to oxidative stress and heat shock (Dinamarco et al., 2012), thus displaying some level of functional conservation as well, despite not showing a significant homology.

### Phenotypic switching regulators

Another virulence factor displayed by the yeast *C. albicans* is the stochastic phenotypic switch known as white-opaque transition (Slutsky et al., 1987; Soll, 1992; Lin et al., 2013). The two cell types differ in shape, gene expression profile, virulence features and colony appearance (Zordan et al., 2007). Opaque cells are the sexually competent form of *C. albicans*, as they present a much higher mating efficiency than white cells (Miller and Johnson, 2002). Despite white-opaque switching occurring spontaneously every 10^4^ generations (Rikkerink et al., 1988), it can be induced by specific environmental conditions, such as high CO_2_ concentration, use of GlcNAc as carbon source, genotoxic stresses and oxidative stress (Kolotila and Diamond, 1990; Ramírez-Zavala et al., 2008; Alby and Bennett, 2009; Huang et al., 2010). However, temperature changes from 25°C to 37°C promote the reverse transition, from opaque to white cells (Slutsky et al., 1987; Srikantha and Soll, 1993). The ability to switch between different phenotypes also constitutes and advantageous trait to enhance its adaptation to host environments (Guan and Liu, 2015), thus affecting a variety of virulence traits (Slutsky et al., 1987; Soll, 1992). Opaque cells are able to colonize skin and escape macrophage detection; whereas white cells are more prone to cause bloodstream infections (Kvaal et al., 1999; Lachke et al., 2003; Lohse and Johnson, 2008). Similar phenomena have been described for *C. parapsilosis, C. tropicalis*, and *C. glabrata* (Lachke et al., 2000; Laffey and Butler, 2005; Moralez et al., 2014). Investigating possible homologous proteins in other fungal species (including *C. neoformans* and *A. fumigatus*) can help to unveil putative specialization events in related proteins from organisms not known to stochastically change their phenotype in this manner. Phenotypic switching is mainly controlled in *C. albicans* by Wor1, regarded as master regulator of this pathway (Zordan et al., 2006; Huang et al., 2010). Consistent with the related switching process occurring in *C. parapsilosis* and *C. tropicalis*, one Wor1 homolog was found in each species, encoded by *ORFs* CPAR2_805000 and *CTRG_03345*, respectively. Interestingly, phylome analysis also revealed one *A. fumigatus* Wor1 homolog, encoded by *ORF Afu6g04490*. Additionally, no homologs were found in *C. neoformans*. Despite Wor1 being the master regulator of white-opaque switching in *C. albicans*, other transcriptional regulators are known to be part of the network controlling this phenomenon. As part of the Wor1 regulon, there are two additional transcription factors involved in white-opaque switching: Wor2 and Czf1. The expression of both transcriptional regulators is directly induced by Wor1, while in turn Wor2 and Czf1 both activate Wor1, creating yet another series of positive feedback loops in the opaque state (Zordan et al., 2007). More recently, two new transcription factors designated Wor3 and Wor4 were added to the existing network governing white-opaque switching. Similarly to what is verified for the previously mentioned regulators, the ectopic expression of Wor3 induced white-opaque switching and is correlated with Wor1, Wor2, and Czf1 (Lohse et al., 2013). As a unique feature, Wor3 was proposed as a member of a different family of DNA-binding proteins. As for Wor4, it was found to be located upstream of Wor1, as its ectopic expression is sufficient to induce white-opaque switching (Lohse and Johnson, 2016). The predicted regulon of this newly discovered transcriptional regulator highly correlates with the ones from Wor1 and Wor2, indicating that Wor4 is integrated in the already described network. Taken together, these transcription factors form an integrated regulatory network with each regulator controlling the expression of the others in the phenotypic switching pathway (Lohse and Johnson, 2016). Analyzing the presence of homologous proteins in other fungal species, similar results are obtained for each regulator. Wor2 phylome analysis identified one closely related protein in *C. parapsilosis*, encoded by *ORF* CPAR2_405400. In the case of Czf1 there is one closely related protein in *C. tropicalis*, encoded by *ORF CTRG_03771*. A similar situation is found for Wor3, with predicted homologs in *C. parapsilosis* and *C. tropicalis*, encoded by *ORFs* CPAR2_202450 and *CTRG_00711*, respectively. As for Wor4, one homolog was also identified in each of *C. parapsilosis* and *C. tropicalis*, encoded by *ORFs* CPAR2_808100 and *CTRG_05581*, respectively. Additionally, in *C. albicans*, a relationship between phenotypic switching and filamentous growth regulators was uncovered, as in white cells Efg1 represses Wor1 in a Wor2-dependent manner. At the same time, in opaque cells Wor1, Wor2, and Czf1 were found to repress Efg1 (Zordan et al., 2007; Lin et al., 2013).

### Iron response regulators

Iron availability is also known to play a crucial role in the establishment of infection. Hosts resist microbial infection by maintaining a low level of free iron to restrict pathogen growth (Hsu et al., 2011). This is achieved by producing transferrin or lactoferrin (Schrettl and Haas, 2011). Therefore, the capacity of a certain pathogen to invade the host is also dependent on its ability to overcome iron deprivation and express iron uptake systems. Among iron uptake and homeostasis regulators, transcription factors belonging to the Hap family can be found in more than one fungal or yeast species. One of the best characterized cases is *A. fumigatus* HapX, a bZIP negative regulator of iron-consuming pathways (e.g., heme biosynthesis, respiration, TCA cycle and amino acid metabolism) that is required for adaptation to iron depletion, while acting as an activator of the siderophore iron uptake pathway, a known virulence factor (Schrettl et al., 2010). Consequently, the HapX mediated iron limitation stress response was interlinked to primary metabolism, oxidative stress and virulence (Brown and Goldman, 2016). Unveiling a more complex role in the regulation of iron homeostasis, HapX was also described to be involved in response to iron excess (Gsaller et al., 2014). Additionally, it was found to be activated by the previously referred regulator SrbA during hypoxia (Blatzer et al., 2011). These observations are in accordance with one of its possible orthologs in *C. glabrata*, Yap5, also described to play a role in both iron excess and iron deprivation conditions (Merhej et al., 2015, 2016). This knowledge indicates a wide-spread role of these regulators in iron sensing, acting as both activators and repressors of gene expression according to differential iron availability. Moreover, in *C. glabrata*, the activation of iron uptake in iron limiting conditions seems to involve the Aft1 transcription factor (*ORF CAGL0H03487g*), as in *S. cerevisiae* (Srivastava et al., 2015). Searching for possible homologs by phylome analysis, no HapX homolog was predicted in the remaining species. However, *C. albicans* harbors the Hap43 transcription factor, a bZIP negative regulator required for low iron response. Such as *A. fumigatus* HapX, Hap43 is responsible for the repression of genes involved in iron-dependent pathways involved in mitochondrial respiration and iron-sulfur cluster assembly (Hsu et al., 2011). Additionally, its role in the regulation of iron acquisition under low iron conditions seems to be more complex, given its action as a positive regulator in iron-limiting conditions (Hsu et al., 2011; Singh et al., 2011). Due to functional conservation, phylome analysis was also performed for *C. albicans* Hap43. As a result, one uncharacterized *C. parapsilosis* protein, encoded by *ORF* CPAR2_209090, was predicted to be a Hap43 homolog (Merhej et al., 2016). Likewise, BLASTp predicts a highly homologous protein in *C. tropicalis* encoded by *ORF CTRG_04121*. In a complementary approach, one of the best studied cases of iron homeostasis in iron replete conditions is the *A. fumigatus* negative regulator SreA (Schrettl et al., 2008). SreA is a GATA transcription factor that negatively regulates siderophore biosynthesis and other iron acquisition genes in the presence of high iron concentrations, including the iron permease FtrA, the ferroxidase FetC and the siderophore-biosynthetic protein SidA (Schrettl et al., 2008). Interestingly, SreA also negatively regulates the previously referred HapX transcription factor (Blatzer et al., 2011). Searching for possible related proteins in other species, no homologous proteins were predicted by phylome analysis. Nevertheless, *C. albicans* Sfu1 is a nice candidate function-wise, playing a function similar to that of SreA. The regulatory relationship observed between *A. fumigatus* SreA and HapX is maintained in *C. albicans* by the negative regulation of Sfu1 over Hap43 (Hsu et al., 2011). In fact, reciprocal Sfu1 phylome analysis revealed *A. fumigatus* SreA as a predicted homolog. Beyond SreA, uncharacterized homologs were also identified in *C. parapsilosis* and *C. tropicalis*, encoded by *ORFs* CPAR2_700810 and *CTRG_03356*, respectively.

### Host immune evasion transcription regulators

Upon infection, human pathogens encounter several barriers that need to be overcome, such as tissue barriers and immune responses. In order to establish infection, fungal pathogens take advantage of the virulence traits analyzed so far, but such traits also include evading the host's cellular immune response. Fungal pathogens display diverse immune evasion strategies, including antigen masking to avoid recognition and persistence/active escaping from phagocytic cells (Netea et al., 2006; Erwig and Gow, 2016). Depending on the strategy applied by each pathogen, distinct sets of genes need to be expressed, uncovering complex regulatory networks according to different environmental conditions.

It should be noted that an additional immune evasion mechanism is known to occur in the yeast *C. albicans*. The capacity of this yeast to undergo yeast-to-hyphae transition, whose regulation is discussed and analyzed in the “Biofilm formation and tissue invasion” section, is described to be an active mechanism for macrophage rupture and evasion after phagocytosis (McKenzie et al., 2010; Lewis et al., 2012a; Rudkin et al., 2013; Bain et al., 2014), and to inhibit macrophage cell division during mitosis (Lewis et al., 2012b).

### Oxidative stress regulators

**Figure 5:**
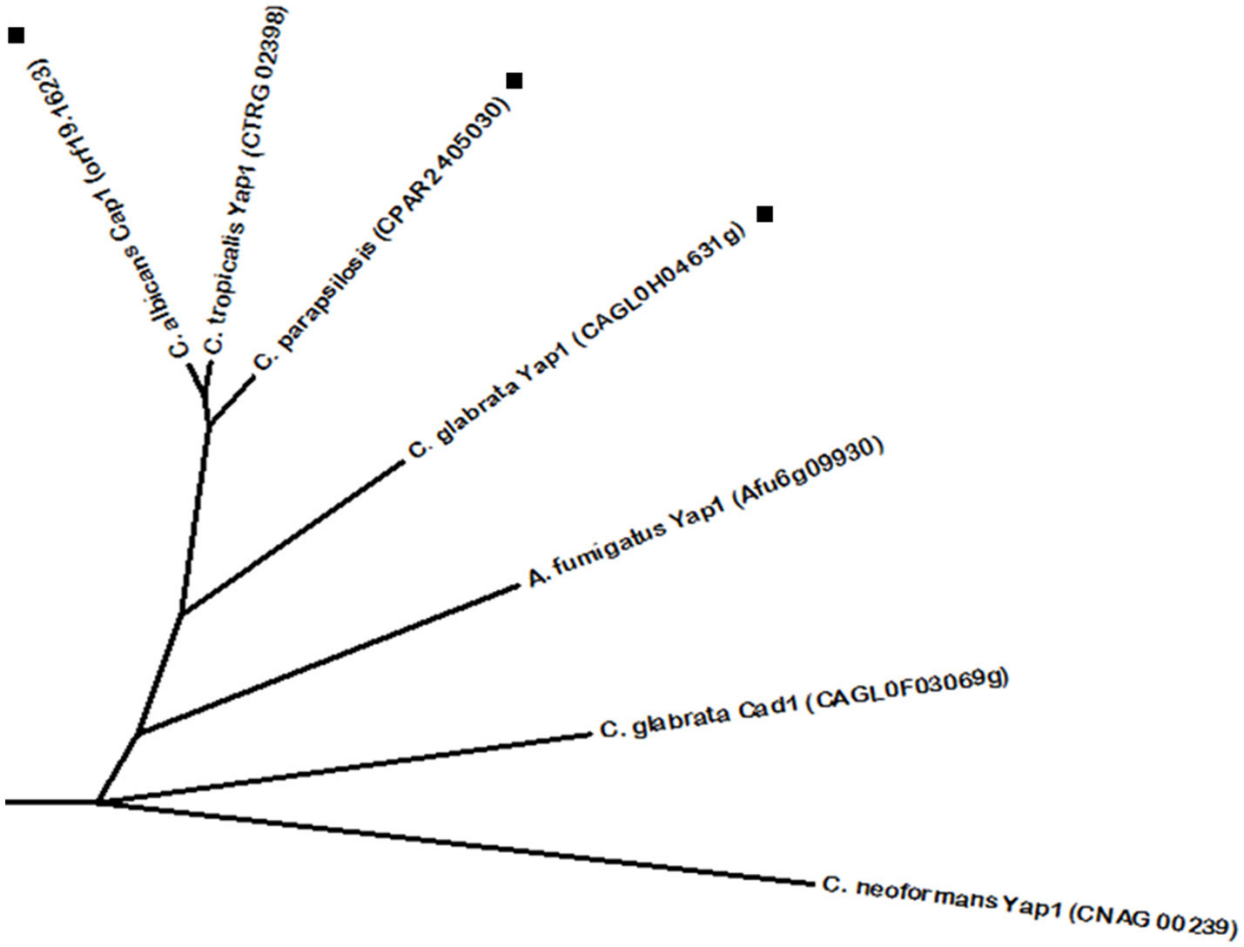
**Phylogenetic analysis of the ***C. albicans*** Cap1 homologs**. Phylome predicted homologs of Cap1 are marked with (■). Unmarked branches represent additional proteins showing some degree of similarity identified by BLASTp (*E* < 10^−50^). The tree was constructed using the Molecular Evolutionary Genetics Analysis (MEGA 7) software (Kumar et al., 2016). Multiple alignments of the amino acid sequences were calculated by ClustalW algorithm (Sneath and Sokal, 1973). The tree is drawn to scale, with branch lengths in the same units as those of the evolutionary distances used to infer the phylogenetic tree. The evolutionary distances were computed using the JTT matrix-based method (Jones et al., 1992) and are in the units of the number of amino acid substitutions per site. The rate variation among sites was modeled with a gamma distribution (shape parameter = 1).

When the immune system response is activated, macrophages, neutrophils, and other phagocytic cells act against fungal pathogens by producing high levels of reactive oxygen species (ROS) and nitric oxide (NO), which results in oxidative and nitrosative stress, respectively (Brown et al., 2009). For this reason, the activation of anti-oxidant responses is a prime strategy upon internalization by phagocytes. The *C. albicans* bZIP regulator Cap1 is one of the most well characterized AP1-like transcription factors. It is responsible for the activation of antioxidant systems, carbohydrate metabolism and energy generation (Limjindaporn et al., 2003). Within its action in defense against ROS, Cap1 directly activates several genes from distinct pathways of antioxidant scavenging, including glutathione S-transferase reactions, superoxide dismutases and the Cat1 catalase (Enjalbert et al., 2006; Wang et al., 2006). As for carbohydrate and energy metabolism, Cap1 upregulates the expression of enzymes involved in NADPH production, which is involved in several redox cycles against ROS (Müller, 2004; Pócsi et al., 2004). Furthermore, a cluster of mitochondrial respiratory genes are expressed in a Cap1-dependent manner, which is consistent with the knowledge that mitochondrial function is required for oxidative stress tolerance in yeast (Grant et al., 1997). Additionally, Cap1 was found to be involved in drug resistance, as it regulates the expression of the multidrug transporter Mdr1 (Mogavero et al., 2011). As depicted in Figure 5, phylome analysis has identified one *C. parapsilosis* protein and *C. glabrata* Yap1 as phylogenetically related to *C. albicans* Cap1. Accordingly, *C. glabrata* Yap1 also has a conserved role in response to oxidative stress, being involved in the induction of conserved genes encoding antioxidant effectors, such as the Cta1 catalase (Cuéllar-Cruz et al., 2008; Roetzer et al., 2011). Just as its *C. albicans* ortholog, *C. glabrata* Yap1 also regulates the expression of the multidrug transporter Flr1, ortholog of the *C. albicans* multidrug transporter Mdr1, particularly in response to 4-nitroquinoline-N-oxide (4-NQO), benomyl and cadmium chloride (Chen et al., 2007). However, there is no evidence to suggest regulation of Flr1 by Yap1 in azole drug resistance (Chen et al., 2007). *C. glabrata* Yap1 has a paralog, Cad1, which was recently found to bind the promoter region of the cadmium response Tna1 protein and the vacuolar transporter Ycf1; however, no changes were detected in Ycf1 expression upon deletion of Cad1; nor any transcriptome changes in response to cadmium (Merhej et al., 2016). Interestingly, *C. tropicalis* also encodes a Yap1 protein, found to be closely related to its *CTG* clade counterparts. Likewise, *A. fumigatus* also possesses a Yap1 transcription factor that despite being encoded by a filamentous fungus is more similar to the proteins from *Candida* spp., than the Yap1 protein from *C. neoformans*. As for the *A. fumigatus* ROS sensing transcription factor Yap1, it was also described to regulate catalase gene expression (*cta1* and *cta2*) and the thioredoxin antioxidant pathway, thus protecting against neutrophil killing (Lessing et al., 2007; Leal et al., 2012). Likewise, *C. neoformans* Yap1 is also involved in the regulation of antioxidant genes, such as thioredoxins and glutathione peroxidases (Paul et al., 2015). Accordingly, the thioredoxin system is known to be required for the response against oxidative stress. It is composed by two thioredoxin proteins and one thioredoxin reductase. The absence of these two thioredoxin proteins, Trx1 and Trx2, leads to growth defect and sensitivity to multiple stresses, while Trx2 is especially important for nitric oxide stress. These findings highlight a high degree of function conservation among Yap1 proteins in several fungal species.

**Figure 6:**
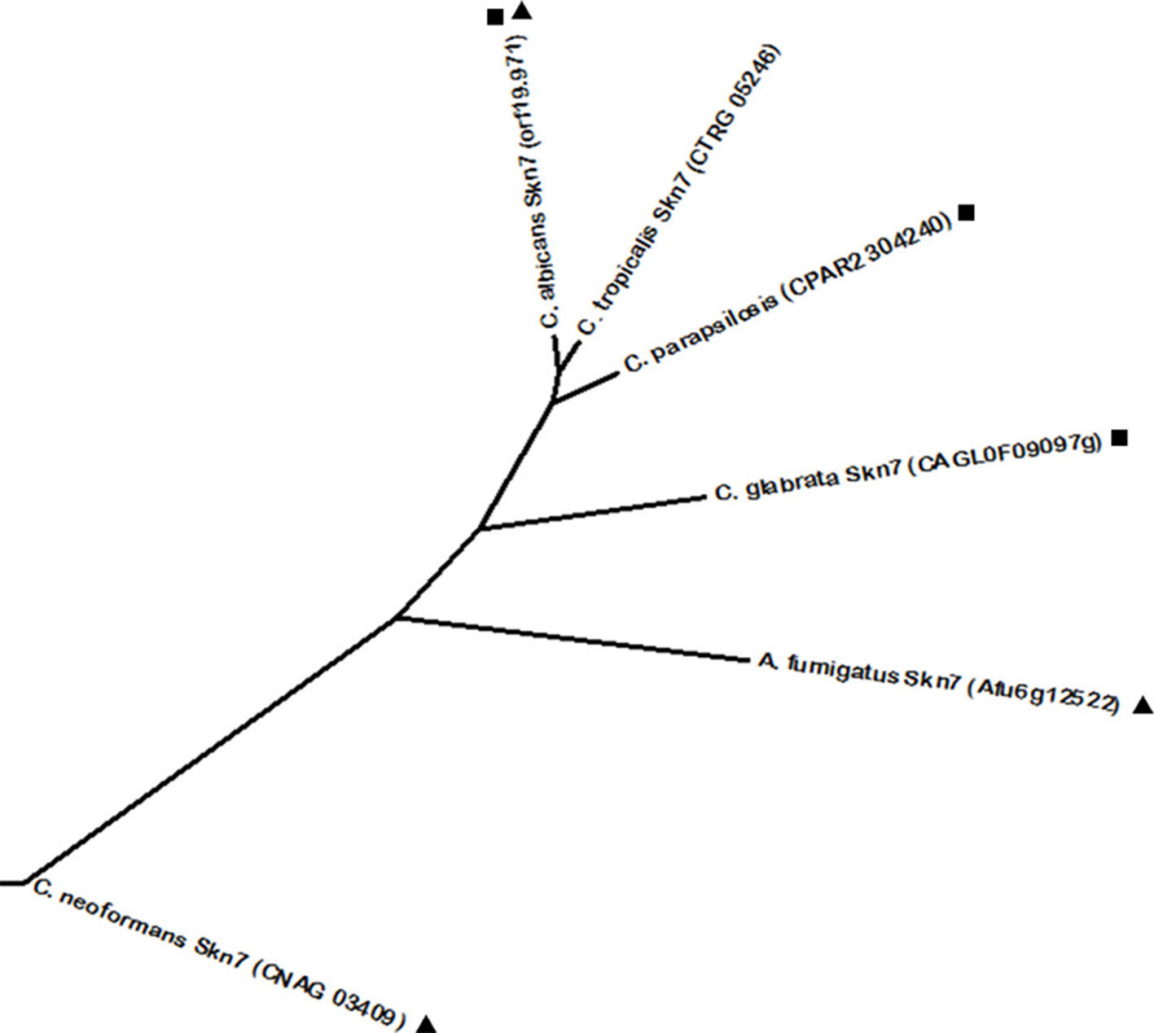
**Phylogenetic analysis of the ***C. albicans*** Skn7 and *C. neoformans* Skn7 homologs**. Phylome predicted homologs of *C. albicans* Skn7 are marked with (■). Phylome predicted homologs of *C. neoformans* Skn7 are marked with (▲). Unmarked branches represent additional proteins showing some degree of similarity identified by BLASTp (*E* < 10^−50^). The tree was constructed using the Molecular Evolutionary Genetics Analysis (MEGA 7) software (Kumar et al., 2016). Multiple alignments of the amino acid sequences were calculated by ClustalW algorithm (Sneath and Sokal, 1973). The tree is drawn to scale, with branch lengths in the same units as those of the evolutionary distances used to infer the phylogenetic tree. The evolutionary distances were computed using the JTT matrix-based method (Jones et al., 1992) and are in the units of the number of amino acid substitutions per site. The rate variation among sites was modeled with a gamma distribution (shape parameter = 1).

Another conserved regulator of oxidative stress resistance in fungi is Skn7. *C. albicans* Skn7 was described as required for hydrogen peroxide resistance in a phenotypic screening (Homann et al., 2009). Phylome analysis revealed as Skn7 predicted homologs one uncharacterized protein in *C. parapsilosis* (encoded by *ORF* CPAR2_304240) and the *C. glabrata* Skn7 (Figure 6). *C. glabrata* Skn7 is involved in hydrogen peroxide response by inducing the expression of the thioredoxins Trx2, Trr1, Tsa1, and the catalase Cta1 (Cuéllar-Cruz et al., 2008; Saijo et al., 2010). Interestingly, there is interdependence of both *C. glabrata* Yap1 and Skn7 over the regulation of a set of genes—such as *TRR1, GPX2, PKH2, TSA1*, and *CTA1*. Additionally, besides Yap1 and Skn7, the transcription factors Msn2 and Msn4 are also involved in the regulation of oxidative stress through regulation of the Cta1, in a concerted action between these four transcription factors (Cuéllar-Cruz et al., 2008). The described interplay of regulatory networks suggests that *C. glabrata* expression of oxidative stress protective genes is well adapted for when it faces a host-pathogen interaction (Roetzer et al., 2011). The *C. tropicalis* Skn7 transcription factor was found to be closely related to its CTG clade homologs, which is reinforced by a high sequence homology determined by BLASTp. Furthermore, *A. fumigatus* and *C. neoformans* also express Skn7 transcription factors. Interestingly, reciprocal phylome analysis revealed a phylogenetic relationship between these regulators and the Skn7 protein from *C. albicans*. Curiously, *A. fumigatus* Skn7 was found to be closer to the remaining yeast proteins than *C. neoformans* Skn7, located significantly further away from the remaining proteins. This is probably due to the fact that while *A. fumigatus* Skn7 has a conserved role in mediating resistance to peroxides (Lamarre et al., 2007), *C. neoformans* Skn7 was found to diverge from the remaining proteins as it specialized in the *C. neoformans* specific trait of melanin production through the activation of the *LAC1* gene (Jung et al., 2015). Melanin accumulates in the cell wall of *C. neoformans* having a protective role against oxidative and temperature stresses. In fact, melanin is an effective antioxidant, protecting *C. neoformans* cells against oxygen and nitrogen oxidants (Wang and Casadevall, 1994). Nevertheless, all Skn7 proteins have a conserved heat shock factor (HSF) DNA binding domain.

Other than the Yap1 and Skn7 families, there are additional less studied regulators of oxidative stress response in *A. fumigatus* and *C. neoformans*, AtfA and Atf1, respectively. AtfA is a transcription factor that targets antioxidant-related genes such as catalase encoding *catA* and dehydrin-like encoding *dprA*, which mediate cellular defense against oxidative stress (Hagiwara et al., 2014). This transcription factor has been also identified in *Saccharomyces* species, but its role in *A. fumigatus* needs further assessment (Hong et al., 2013). As for Atf1, it is described to be required for oxidative stress induction of the thioredoxin genes *TRX1* and *TRX2* in *C. neoformans* (Missall and Lodge, 2005). Interestingly, Atf1 was also found to repress melanin and capsule formation, as null Atf1 mutants show increased capsule and melanin production. Since Atf1 is regulated by Can2, Pka1, and Rim101, it is possible that once again the cAMP pathway might be involved in this network (Kim et al., 2010). Additionally, Atf1 was also found to play a role in thermotolerance and drug resistance, given that its absence was seen to increase resistance against amphotericin B and fluconazole (Kim et al., 2010). In this case, phylome analysis predicted AtfA and Atf1 as homologs, thus showing a high degree of sequence similarity, translated in their functional conservation. Curiously, the Sko1 transcription factor from *C. albicans* was found to share sequence similarity with *A. fumigatus* AtfA, by phylome analysis. Given this finding, other potential Sko1 proteins were searched in other *Candida* spp. Sko1 phylome predicted one uncharacterized *C. tropicalis* homolog (encoded by *ORF CTRG_04352*) and the *C. glabrata* Sko1 protein. All proteins contain a bZIP domain, but given the knowledge concerning *C. albicans* Sko1 it is possible that these proteins have a participation in additional roles (e.g., cell wall stress and virulence) while still maintaining activity in oxidative stress response, as it was described for *C. albicans* Sko1 control over the dehydrogenase Ifd4 (Alonso-Monge et al., 2010; Singh et al., 2011).

### Nitrosative stress regulators

In what concerns resistance to nitrosative stress, one of the best studied cases is the *C. albicans* regulator Cta4, a Zn(2)-Cys(6) zinc finger positive regulator of nitrosative stress response. It is upregulated upon nitric oxide exposure and it was found to be required for the expression of the nitric oxide dioxygenase Yhb1, required for NO consumption and detoxification, by directly binding the regulatory region of its gene (Ullmann et al., 2004; Chiranand et al., 2008). In *C. glabrata*, the transcription factor Yap7 was shown to exert control over Yhb1 by strongly inhibiting its expression in a direct manner; a regulatory association also verified in *S. cerevisiae* (Merhej et al., 2015). Another described player in nitrosative stress response in pathogenic yeasts is *C. neoformans* Yap4, an activator of the thioredoxin genes in *C. neoformans* in response to nitrosative stress, especially Trx2 (Missall and Lodge, 2005). Just as the Yap1 case, *C. neoformans* Yap4 contains a bZIP domain, however, Yap4 phylome did not reveal any homologs in the studied species. Nevertheless, there are known Yap4 proteins in some *Candida* spp., namely *C. albicans* Cap4 and *C. glabrata* Yap4/6. These proteins remain uncharacterized, therefore it would be interesting to assess if their role has diverged, given that no relationship was identified with the nitrosative stress regulator *C. neoformans* Yap4. Using phylome analysis, homology relationships among *C. albicans* Cap4, *C. glabrata* Yap4/6 and the *C. parapsilosis* protein encoded by *ORF* CPAR2_11470 were identified.

### Amino acid starvation regulators

One additional factor also thought to play a role in phagocyte persistence and evasion by pathogenic yeasts is the reprogramming of carbohydrate and amino acid metabolism. This has to do with the fact that the environment present inside phagocytic cells is often limiting in terms of nutrients, especially nitrogen sources (Pérez-delos Santos and Riego-Ruiz, 2016). In this context, the bZIP transcription factor Gcn4 was identified in *C. albicans* as playing a key role in amino acid control response and was found to be expressed upon neutrophil phagocytosis (Fradin et al., 2005). It activates the transcription of amino acid biosynthetic genes (*HIS4, HIS7, LYS1, LYS2*, and *ARO4*) via Gcn4-response elements (GCRE) (Tripathi et al., 2002). Among others, Gcn4 plays a role in the biosynthetic pathway of arginine, which in turn is involved in the production of CO_2_ and urea; products that induce filamentation inside macrophages as an escape mechanism and neutralization of the acidic pH of the phagolysosome (Ghosh et al., 2009; Vylkova et al., 2011). This is further supported by the observation that Gcn4 is required for Efg1-dependent filament induction by amino acid starvation, but not by serum (Tripathi et al., 2002). *C. albicans* Gcn4 is closely related to proteins found in other CTG clade species, including a *C. tropicalis* protein encoded by *ORF CTRG_02060* and a *C. parapsilosis* Gcn4 protein. Additionally, phylome analysis also revealed the *C. glabrata* Gcn4 regulator as being closely related. Moreover, *A. fumigatus* harbors a regulator with a related function: CpcA. Despite not showing enough similarity with Gcn4, CpcA was also found to be a transcriptional activator of amino acid biosynthesis and to play a role in virulence in this filamentous fungus (Krappmann et al., 2004).

### Gliotoxin and melanin production regulators

An additional host immune evasion mechanism is the production of toxins by some pathogens. This is particularly true for *A. fumigatus*, in which the C_2_H_2_ zinc finger containing transcription factor MtfA is involved in the expression of gliotoxin genes, *gliZ* and *gliP*, and its biosynthesis, as well as protease activity in the secretome and conidiation (Smith and Calvo, 2014). Gliotoxins have been described as possessing anti-inflammatory and immunosuppressive activities toward the host immune effector cells, including neutrophils and macrophages (Stanzani et al., 2005; Orciuolo et al., 2007; Scharf et al., 2012). No homologs in the additional fungal pathogens considered in this review are predicted by phylome analysis, which can be correlated with the specialized function of MtfA in gliotoxin production regulation.

Another important immune evasion mechanism displayed in *C. neoformans* is melanin synthesis, which is possible in the presence of exogenous dihydroxyphenols, catalyzed by a phenoloxidase (Almeida et al., 2015). Once yeast cells are phagocytosed, the production of melanin protects cells against the oxidative environment inside the phagolysosome (Panepinto and Williamson, 2006). According to a systematic functional profiling analysis, several transcription factors are assumed to be involved in the regulation of melanin production, including Mbs1 (Jung et al., 2015), however the particular pathways and role it regulates are still unknown. Despite melanin production is a specific trait of neurotropic fungi, such as *C. neoformans*, and is not known to occur in *Candida* spp. or *A. fumigatus*, investigating possible homologous proteins in other fungal species can help to shed light in this regard by unveilling putative related roles. Evaluating possible homology relationships of *C. neoformans* Mbs1 with other species, *C. albicans* Mbp1, Swi4 and Swi6 were identified as homologs by phylome analysis, despite not appearing to have a conserved function, given their involvement in G1/S cell cycle progression (Côte et al., 2009; Hussein et al., 2011). Additionally, one uncharacterized *A. fumigatus* protein, encoded by *ORF Afu7g05620*, was also identified as a homolog. Interestingly, one additional *C. neoformans* protein, encoded by *ORF* CND05520, was found to be phylogenetically related to Mbs1. Also, BLASTp unveiled the *A. fumigatus* protein encoded by *ORF Afu3g13920* as a possible homolog. As referred, *C. albicans* Mbp1 was found to share significant similarity with *C. neoformans* Mbs1. Reciprocal phylome analysis did not reveal *C. neoformans* Mbs1 as a predicted homolog, instead, four proteins within *Candida* spp. were predicted as homologs, namely *C. tropicalis* Mbp1, a *C. parapsilosis* protein encoded by *ORF* CPAR2_102740 and two *C. glabrata* proteins encoded by *ORFs CAGL0D01012g* and *CAGL0A04565g*. Other than *Candida* spp. homologs, the same *A. fumigatus* protein found to share homology with *C. neoformans* Mbs1 was also identified to be related to *C. albicans* Mbp1 by reciprocal phylome analysis.

## Conclusions and future perspectives

The study of drug resistance, virulence and immune evasion mechanisms in fungal pathogens has advanced considerably over the past years. However, the transcriptional control of such mechanisms has been studied to a different extent. The most studied transcriptional regulators in *A. fumigatus, C. neoformans*, and *Candida* spp. are those relevant to their virulence features. Nonetheless, this review highlights that major transcriptional regulators vary among species, leading to disperse information regarding regulatory networks in each pathogen.

**Figure 7:**
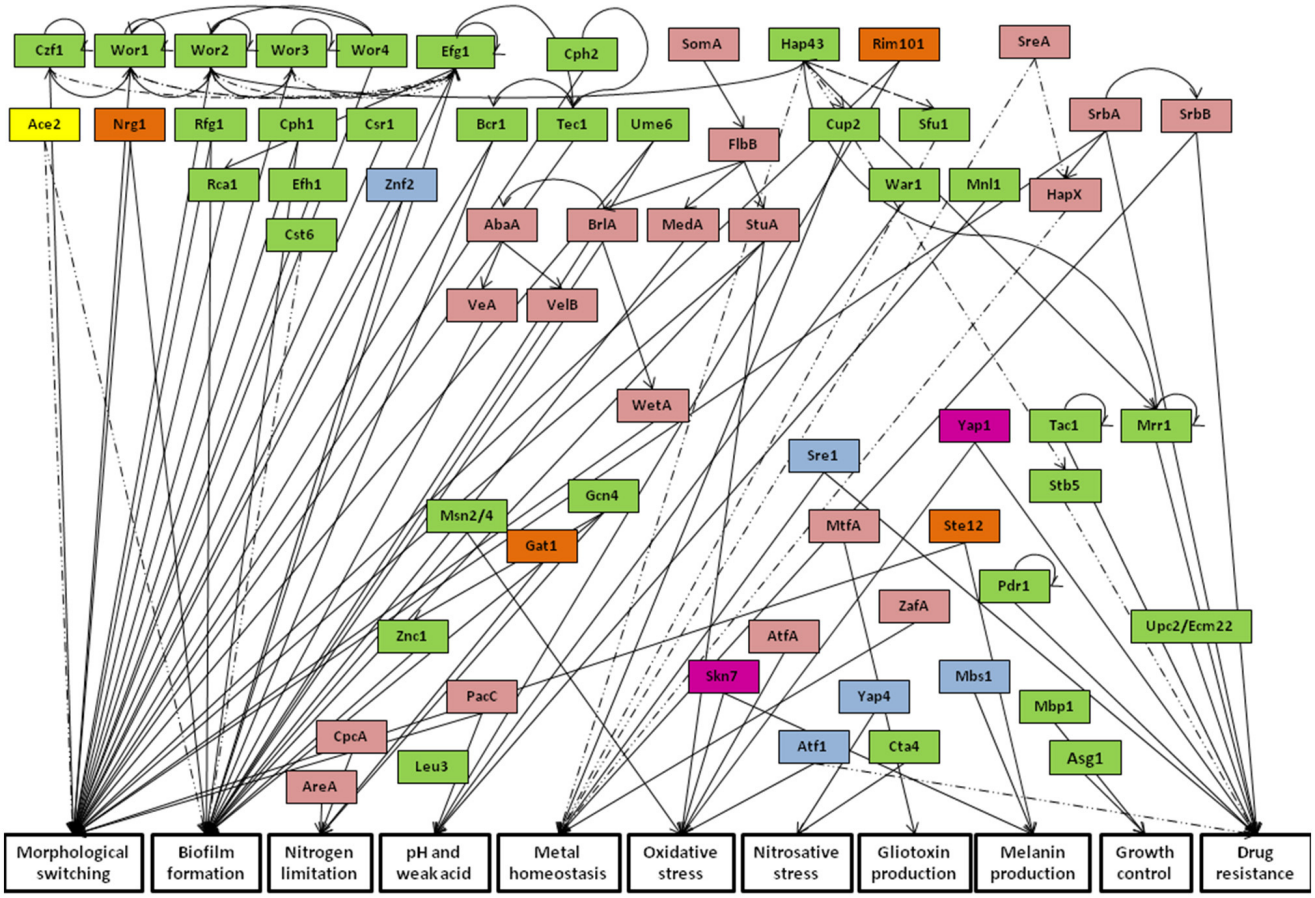
**Transcriptional networks of described transcription factors among ***A. fumigatus***, ***C. neoformans*** and ***Candida*** spp. Transcriptional factors regulating distinct biological mechanisms are shown**. Full lines represent positive regulation, dotted lines represent negative regulation. Colored boxes indicate the species that include characterized homologs of the indicated transcription factor, according to the following color code: Single species: Green, *Candida* spp.; Pink, *A. fumigatus*; Blue, *C. neoformans*; Multiple species: Orange, *Candida* spp. + *C. neoformans*; Yellow, *Candida* spp. + *A. fumigatus*; Purple, *Candida* spp. + *A. fumigatus* + *C. neoformans*.

The regulatory networks analyzed in this review are compiled in Figure 7. It is possible to observe that only the Yap1 and Skn7 transcription factors, involved in the response to oxidative stress, are conserved with similar functions in all the fungal pathogens considered in this review (Figure 7, purple). This fact points out to the primordial importance of being able to fight reactive oxygen species inside the host, and specifically, within macrophages, something that is common to all pathogens. Other transcription factors, such as Ace2, Ste12, Nrg1, Gat1, Upc2/Ecm22, and Rim101, were also found in most, but not all, of the considered species (Figure 7, yellow and orange), playing important roles in morphological switching, nutrient homeostasis, drug resistance and nitrosative and pH stress tolerance. It is interesting to point out, however, that most of the characterized transcription factors are specific for *Candida* species (Figure 7, green), *C. neoformans* (Figure 7, blue) or *A. fumigatus* (Figure 7, pink), an observation that highlights the divergence of regulatory control in these different fungal pathogens, suggesting that different molecular mechanisms have been adopted to tackle similar biological needs.

Another interesting feature that comes from the observation of Figure 7 is that, in general, transcriptional control of a given mechanism is controlled by one or a few major regulators, that may also play complementary roles in other biological process. Examples of these occurences are transcription factors involved in morphology changes that are also often associated with biofilm formation, given that the two processes are associated. Common regulatory networks are also found in the establishment of biofilms and resistance to oxidative stress or drug resistance. The existence of regulatory circuits in which transcription factors do not regulate directly effector genes, but rather other transcription factors is also commonly verified. All these aspects translate complex intricated circuits with a series of interconnect relationships among regulators and the biological processes regulated by them. Regulatory network architecture is significantly complex, comprising multiple layers of regulation among expression control of transcription factors, effector genes and environmental conditions.

Differential knowledge concerning transcriptional control can be associated with the occurence of specific traits not known to occur in every fungal pathogen. These include gliotoxin production in filamentous fungi such as *A. fumigatus* and melanin production in neurotropic fungi such as *C. neoformans*.

The occurence of known or predicted homologous regulators among the different fungal pathogens considered in this review and the corresponding degree of functional conservation in infection-related processes is summarized in Table 1. To be highlighted is the presence of conserved transcriptional regulators that present divergent roles among species, participating in distinct mechanisms and regulating distinct targets. Examples of such ocurrences are the cases of Mbs1 and ZafA, which acquired new functions in *C. neoformans* and *A. fumigatus*, respectively, in comparison to their homologs. In some cases, this situation represents a specialization of a given protein to pathogen-specific pathway. The inverse situation is also observed, where distinct regulators in different species are found to participate in the control of similar features, such as the *C. albicans* Tac1 and the *C. glabrata* Pdr1 transcription factors, or *C. albicans* Efh1 and *A. fumigatus* StuA.

**Table 1:** **Conservation of function between described transcription factors in ***A. fumigatus***, ***C. neoformans*** and ***Candida*** spp. and their correspondent homologs predicted by the Phylome DB**.

**Function**	**Transcription factor**	**Similar function (homologs)**	**Distinct function (homologs)**	**Unknown function (homologs)**	**Similar function (non-homologs)**
Drug resistance (efflux pumps expression)	Pdr1 (*C. glabrata*)	N.A.	N.A.	N.A.	Tac1 (*C. albicans*)
					Mrr1 (*C. albicans*)
					Mrr2 (*C. albicans*)
	Stb5 (*C. glabrata*)	Stb5 (*C. albicans*)	N.A.	CPAR2_109760; CTRG_04421^*^	N.A.
	Tac1 (*C. albicans*)	N.A.	Znc1 (*C. albicans*)	CPAR2_303510; CPAR2_303520	Pdr1 (*C. glabrata*)
				CPAR2_303500; *CTRG_05307*	Mrr1 (*C. albicans*)
			Hal9 (*C. albicans*)	*CTRG_05306*; *CTRG_05308*	Mrr2 (*C. albicans*)
	Mrr1 (*C. albicans*)	Mrr1 (*C. parapsilosis*)	Cta4 (*C. albicans*)	orf19.5133; orf19.7371	Pdr1 (*C. glabrata*)
				CPAR2_501570; CPAR2_405260 CPAR2_405270; CPAR2_704130	Tac1 (*C. albicans*) Mrr2 (*C. albicans*)
				CPAR2_807260; CTRG_02269^*^	
				CTRG_02696^*^; CTRG_02712^*^	
				CTRG_02268^*^; CTRG_05208^*^	
				CTRG_00538^*^; CTRG_02271^*^	
				CPAR2_501570; CPAR2_405260 CPAR2_405270; CPAR2_704130	
	Mrr2 (*C. albicans*)	Mrr1 (*C. parapsilosis*)	Cta4 (*C. albicans*)	CPAR2_405270; CPAR2_807820	Pdr1 (*C. glabrata*)
				*CAGL0M12298g*; CTRG_05568^*^	Tac1 (*C. albicans*)
					Mrr1 (*C. albicans*)
Drug resistance (ergosterol biosynthesis)	Upc2 (*C. albicans*)	Upc2 (*C. parapsilosis*)	N.A.	Ecm22 (*C. albicans*)	N.A.
		Upc2a (*C. glabrata*)^*^		Upc2 (*C. tropicalis*)^*^	
		Upc2b (*C. glabrata*)^*^			
	SrbA (*A. fumigatus*)	SrbB (*A. fumigatus*)	N.A.	N.A.	Sre1 (*C. neoformans*)
Virulence (hyphal growth/biofilm formation)	Efg1 (*C. albicans*)	Efg1 (*C. parapsilosis*)	StuA (*A. fumigatus*)	*CAGL0L01771g*	N.A.
		StuA (*A. fumigatus*)			
		Efh1 (*C. albicans*)		*CTRG_01780*	
	Efh1 (*C. albicans*)	Efg1 (*C. albicans*)	N.A.	Efh1 (*C. parapsilosis*); *CTRG_01780*	StuA (*A. fumigatus*)
	Cph1 (*C. albicans*)	Ste12 (*C. glabrata*)	N.A.	Cph1 (*C. parapsilosis*)	N.A.
				Cph1 (*C. tropicalis*)^*^	
	Ste12 (*C. glabrata*)	Cph1 (*C. albicans*)	N.A.	*CAGL0H02145g;* SteA (*A. fumigatus*)	N.A.
	Tec1 (*C. albicans*)	N.A.	N.A.	CPAR2_805930	AbaA (*A. fumigatus*)
	Bcr1 (*C. albicans*)	Bcr1 (*C. parapsilosis*)	N.A.	*CTRG_00608*	N.A.
	Cst6 (*C. glabrata*)	N.A.	N.A.	N.A.	Rca1 (*C. albicans*)
	Rca1 (*C. albicans*)	N.A.	N.A.	CPAR2_109540; CTRG_04281^*^	N.A.
	SomA (*A. fumigatus*)	N.A.	N.A.	N.A.	N.A.
	BrlA (*A. fumigatus*)	N.A.	N.A.	N.A.	N.A.
	MedA (*A. fumigatus*)			CNAG_03859^*^	N.A.
	Znf2 (*C. neoformans*)	Csr1 (*C. albicans*)	ZafA (*A. fumigatus*)	N.A.	N.A.
	Nrg1 (*C. albicans*)	Nrg1 (*C. tropicalis*)	N.A.	CPAR2_300790; *CTRG_00608*	N.A.
				*CAGL0G08107g*	
	Csr1 (*C. albicans*)	N.A.	N.A.	CPAR2_403080; *CTRG_03883*	N.A.
				*CAGL0J05060g*	
	Rfg1 (*C. albicans*)	N.A.	N.A.	CPAR2_801100	N.A.
	Ace2 (*C. glabrata*)	Swi5 (*C. glabrata*)	N.A.	N.A.	Ace2 (*C. albicans*)
					Ace2 (*A. fumigatus*)
	Ace2 (*C. albicans*)	Ace2 (*C. parapsilosis*)	N.A.	CTRG_03073^*^	Ace2 (*C. glabrata*)
					Ace2 (*A. fumigatus*)
Virulence (nitrogen use)	Gat1 (*C. albicans*)	N.A.	N.A.	Gat1; CTRG_03831^*^	AreA (*A. fumigatus*)
Virulence (pH and weak acid response)	Rim101 (*C. albicans*)	N.A.	N.A.	CPAR2_700450	PacC (*A. fumigatus*)
				Rim101 (*C. tropicalis*)^*^	
	War1 (*C. albicans*)	N.A.	N.A.	CPAR2_110360; *CTRG_04350*	N.A.
				War1 (*C. glabrata*)	
				*Afu7g01640*; *Afu8g00950*	
	Mnl1 (*C. albicans*)	N.A.	N.A.	CPAR2_204380	Msn4 (*C. albicans*)
					Msn2 (*C. glabrata*)
				*CTRG_03074*	Msn4 (*C. glabrata*)
					SebA (*A. fumigatus*)
	Msn4 (*C. albicans*)	N.A.	N.A.	CPAR2_301730	Mnl1 (*C. albicans*)
					Msn2 (*C. glabrata*)
				*CTRG_03253*	Msn4 (*C. glabrata*)
					SebA (*A. fumigatus*)
Phenotypic switching	Wor1 (*C. albicans*)	N.A.	N.A.	CPAR2_805000; *CTRG_03345*	N.A.
				*Afu6g04490*	
	Wor2 (*C. albicans*)	N.A.	N.A.	CPAR2_405400	N.A.
	Czf1 (*C. albicans*)	N.A.	N.A.	*CTRG_03771*	N.A.
	Wor3 (*C. albicans*)	N.A.	N.A.	CPAR2_202450; *CTRG_00711*	N.A.
	Wor4 (*C. albicans*)	N.A.	N.A.	CPAR2_808100; *CTRG_05581*	N.A.
Iron homeostasis	HapX (*A. fumigatus*)	N.A.	N.A.	N.A.	Hap43 (*C. albicans*)
	Hap43 (*C. albicans*)	N.A.	N.A.	CPAR2_209090; CTRG_04121^*^	N.A.
	SreA (*A. fumigatus*)	Sfu1 (*C. albicans*)	N.A.	N.A.	N.A.
	Sfu1 (*C. albicans*)	N.A.	N.A.	CPAR2_700810; *CTRG_03356*	N.A.
Oxidative stress resistance (antioxidant gene expression)	Cap1 (*C. albicans*)	Yap1 (*C. glabrata*)	N.A.	CPAR2_405030	Yap1 (*A. fumigatus*)
				Yap1 (*C. tropicalis*)^*^	Yap1 (*C. neoformans*)
	Skn7 (*C. albicans*)	Skn7 (*C. glabrata*)	N.A.	CPAR2_304240; Skn7 (*C. tropicalis*)^*^	Skn7 (*A. fumigatus*)
	Skn7 (*A. fumigatus*)	Skn7 (*C. albicans*)	Skn7 (*C. neoformans*)	N.A.	Skn7 (*C. glabrata*)
	AtfA (*A. fumigatus*)	Atf1 (*C. neoformans*)	Atf1 (*C. neoformans*)	N.A.	N.A.
	Sko1 (*C. albicans*)	AtfA (*A. fumigatus*)	N.A.	*CTRG_04352*; Sko1 (*C. glabrata*)	N.A.
Nitrosative stress resistance	Cta4 (*C. albicans*)	N.A.	Mnl1 (*C. albicans*)	N.A.	N.A.
			Msn4 (*C. albicans*)		
	Yap4 (*C. neoformans*)	N.A.	N.A.	N.A.	N.A.
Amino acid starvation	Gcn4 (*C. albicans*)	N.A.	N.A.	Gcn4 (*C. parapsilosis*)	CpcA (*A. fumigatus*)
				*CTRG_02060*; Gcn4 (*C. glabrata*)	
Gliotoxin production	MtfA (*A. fumigatus*)	N.A.	N.A.	N.A.	N.A.
Melanin production	Mbs1 (*C. neoformans*)	N.A.	Mbp1 (*C. albicans*)	Swi6 (*C. albicans*); *Afu7g05620*	N.A.
			Swi4 (*C. albicans*)	Afu3g13920^*^; CND05520	
Cell cycle	Mbp1 (*C. albicans*)	N.A.	N.A.	CPAR2_102740; *CAGL0D01012g*	N.A.
				*CAGL0A04565g*; *Afu7g05620*	
				Mbp1 (*C. tropicalis*)	

N.A., Not Available;

* *Homologs identified by BLASTp*.

The regulation of transcriptional networks is complex and presents significant variations among different fungal pathogens, either in terms of regulators themselves or their regulatory targets. The study of regulatory circuits should therefore be a prime strategy in the fight against fungal infections, allowing to develop better diagnostic and treatment approaches according to each pathogen's conserved or specific pathways.

## Author contributions

PP, CC, MC, and DR reviewed the literature on the manuscript related subject, foccusing on *Candida* species, *Cryptococcus neoformans* and *Aspergillus fumigatus*, respectively. PP and MCT wrote most of the manuscript. MCT was the scientific coordinator of this work.

### Conflict of interest statement

The authors declare that the research was conducted in the absence of any commercial or financial relationships that could be construed as a potential conflict of interest.
